# The Coevolution of Cellularity and Metabolism Following the Origin of Life

**DOI:** 10.1007/s00239-020-09961-1

**Published:** 2020-08-18

**Authors:** Yuta A. Takagi, Diep H. Nguyen, Tom B. Wexler, Aaron D. Goldman

**Affiliations:** 1 Department of Biology, Oberlin College, Oberlin, OH 44074 USA; 2 Computational and Systems Biology, Massachusetts Institute of Technology, Cambridge, MA 02139 USA; 3 Department of Computer Science, Oberlin College, Oberlin, OH 44074 USA; 4 Verily Life Sciences, Cambridge, MA USA; 5 Blue Marble Space Institute of Science, Seattle, WA 98154 USA

**Keywords:** Origin of life, Digital life, Ancient life, Early evolution, Coevolution, Evolution of cellularity, Evolution of metabolism

## Abstract

The emergence of cellular organisms occurred sometime between the origin of life and the evolution of the last universal common ancestor and represents one of the major transitions in evolutionary history. Here we describe a series of artificial life simulations that reveal a close relationship between the evolution of cellularity, the evolution of metabolism, and the richness of the environment. When environments are rich in processing energy, a resource that the digital organisms require to both process their genomes and replicate, populations evolve toward a state of non-cellularity. But when processing energy is not readily available in the environment and organisms must produce their own processing energy from food puzzles, populations always evolve both a proficient metabolism and a high level of cellular impermeability. Even between these two environmental extremes, the population-averaged values of cellular impermeability and metabolic proficiency exhibit a very strong correlation with one another. Further investigations show that non-cellularity is selectively advantageous when environmental processing energy is abundant because it allows organisms to access the available energy, while cellularity is selectively advantageous when environmental processing energy is scarce because it affords organisms the genetic fidelity required to incrementally evolve efficient metabolisms. The selection pressures favoring either non-cellularity or cellularity can be reversed when the environment transitions from one of abundant processing energy to one of scarce processing energy. These results have important implications for when and why cellular organisms evolved following the origin of life.

**Electronic supplementary material:**

The online version of this article (10.1007/s00239-020-09961-1) contains supplementary material, which is available to authorized users.

## Introduction

The cell is, by definition, the basic structural unit of all organisms. Cellular organization defines the boundaries between organisms and their environment, allowing organisms to control their internal chemistry, to generate and store their own chemical potential energy, and to build and replicate complex systems with high fidelity. Cellularity also confers individuality upon organisms by storing genetic material that can be passed on to future progeny through cell replication. This vertical inheritance, made possible by cellular organization, may have been a prerequisite for the first speciation events in early evolutionary history (Woese 1998).

Supporting this notion, strong evidence suggests that the last universal common ancestor of life (Becerra et al. 2007; Goldman et al. 2013) represents a cellular organism or a population of cellular organisms. The genome of the last universal common ancestor, for example, likely encoded a signal recognition particle and receptor system for directing proteins to be embedded within or secreted through the cell membrane (Gribaldo and Cammarano 1998), a process that, today, occurs in the endoplasmic reticulum of eukaryotes, but in the cell membrane of bacteria and archaea. The genome of the last universal common ancestor also very likely encoded the ATP synthase motor (Gogarten and Taiz 1992), which takes advantage of proton gradients across membranes to generate ATP from ADP and phosphate. Finally, the genome of the last universal common ancestor could likely synthesize its own phospholipid membrane constituents. Despite the fact that archaeal phospholipids are composed of isoprenoid ethers while bacterial and eukaryotic phospholipids are composed of fatty acid esters (Kates 1978), this difference appears to result from only a few variant metabolic steps in an overall pathway that is otherwise shared between bacteria and archaea (Peretó et al. 2004).

The last universal common ancestor, though very ancient, was the product of many evolutionary transitions following the origin of life (Maynard and Szathmáry and 1995; Szathmáry and Maynard 1995; Poole et al. 1998; Goldman et al. 2010; Goldman and Landweber 2012; Petrov et al. 2015). The origin of life likely began, not as a cellular organism, but as a simple genetic or chemical replicator. The RNA world hypothesis (Gilbert 1986) proposes that life began as a set of catalytic RNAs or pre-RNAs that were capable of replicating themselves through polymerase-like enzymatic activity (Johnston et al. 2001; Wochner et al. 2011). Other hypotheses about the origin of life propose that life arose from autocatalytic sets of catalysts that, as a whole, could replicate the entire catalytic network (Kauffman 1971). Recent work suggests that these two frameworks for understanding the origin of life are neither complete nor are they mutually exclusive, with many arguing that an RNA world could only have existed within a broader chemical context (Bowman et al. 2015; Goldman et al. 2016) and others showing that catalytic RNAs can, themselves, form cooperative autocatalytic networks (Vaidya et al. 2012).

Even though life likely began as a simple system of genetic or chemical replicators, naturally occurring membranes may have also played an important role in the origin of life. Membranes have been demonstrated to self-assemble from prebiotically plausible compounds such as decanoic acid (Namani and Walde 2005) and can even self-assemble from the lipid fractions of carbonaceous chondrite meteors (Deamer and Pashley 1989), suggesting that membrane structures may have been present during the origin of life. Model protocells, which are meant to mimic membrane structures following the origin of life but prior to the origin of true cellularity, are capable of growth and division (Zhu and Szostak 2009) and have been shown to concentrate and stabilize compounds that may have been important during and after the origin of life (Adamala and Szostak 2013; Black et al. 2013).

Alongside these chemistry-oriented accounts of the behavior of membranes and protocells during and directly after the origin of life, a number of plausible evolutionary hypotheses have been developed to better understand the selection pressures under which true cellularity may have evolved following the origin of life. One early hypothesis suggested that selection on groups of genes housed within a cell could have counterbalanced the deleterious effects of internal selection between genes, thus stabilizing a gene complement composed of multiple genes and gene functions (Eigen and Schuster 1977; Szathmáry and Maynard 1995). In addition, cellular boundaries could potentially act to exclude selfish replicators (Eigen et al. 1981; Goldman and Landweber 2012) and cellular organization may have also selected for the chromosomal linkage of genes (Maynard and Szathmáry 1993), which could further alleviate inter-gene conflict (Szathmáry 2015). Recent computational modeling studies have shown that spatial self-organization without compartmentalization can provide similar genetic stability (Takeuchi and Hogeweg 2009; Hogeweg and Takeuchi 2003), but that protocell compartmentalization is superior to spatial self-organization because it can better mitigate the effects of parasites and can accommodate more extreme mutation rates through the cooperative function of polymerases (Shah et al. 2019). Recent microfluidics experiments have confirmed these modeling results by demonstrating that even transient compartmentalization can protect RNA replicators from parasites (Matsumura et al. 2016).

In addition to these genetic considerations, several hypotheses propose that metabolic factors influenced the early evolution of membranes and protocells. One general proposal by Szathmáry (2007) suggests that early membranes were permeable to metabolites and served only to organize the genetic material, but as enzyme-mediated metabolism became increasingly sophisticated, membranes coevolved alongside metabolism to be more selective and to further differentiate the internal and external chemical environments. This regulation of the chemical environment may have, in turn, allowed organisms to colonize environments beyond the geochemical setting in which life originated (Cantine and Fournier 2018). Another, more specific metabolic hypothesis proposes that cellular membranes evolved as a way to maintain voltage gradients and use them to harness chemical potential energy (Martin and Russell 2003; Lane and Martin 2012), as is seen across extant life in the form of the proton-driven ATP synthase motor. These genetic and metabolic hypotheses are not mutually exclusive and all of them may help explain the evolution of cellularity following the origin of life.

Here, we describe a study in which we investigated selection pressures that favored either cellularity or non-cellularity using an artificial life simulation system in which cellularity is an evolvable trait. The artificial life model is naïve to the hypotheses listed above and allows several different genetic and environmental parameters to be manipulated. Early explorations of the variable parameters suggested that the relationship between cellularity and metabolism was very strong within the context of our model. We did not originally set out to test the Szathmáry hypothesis (2007) that membrane permeability coevolved in response to increasing metabolic capability, but our results provide direct evidence in support of this hypothesis. They also demonstrate that Szathmáry’s metabolic hypothesis is fundamentally linked to those hypotheses that relate cellularity to genetic fidelity. By controlling certain environmental parameters, however, we show that cellularity would have been strongly selected against during the early stages of the origin of life process, suggesting that cellularity only arose after some change in the setting where the origin of life took place.

## Results

### Description of the Artificial Life Simulation

**Fig. 1:**
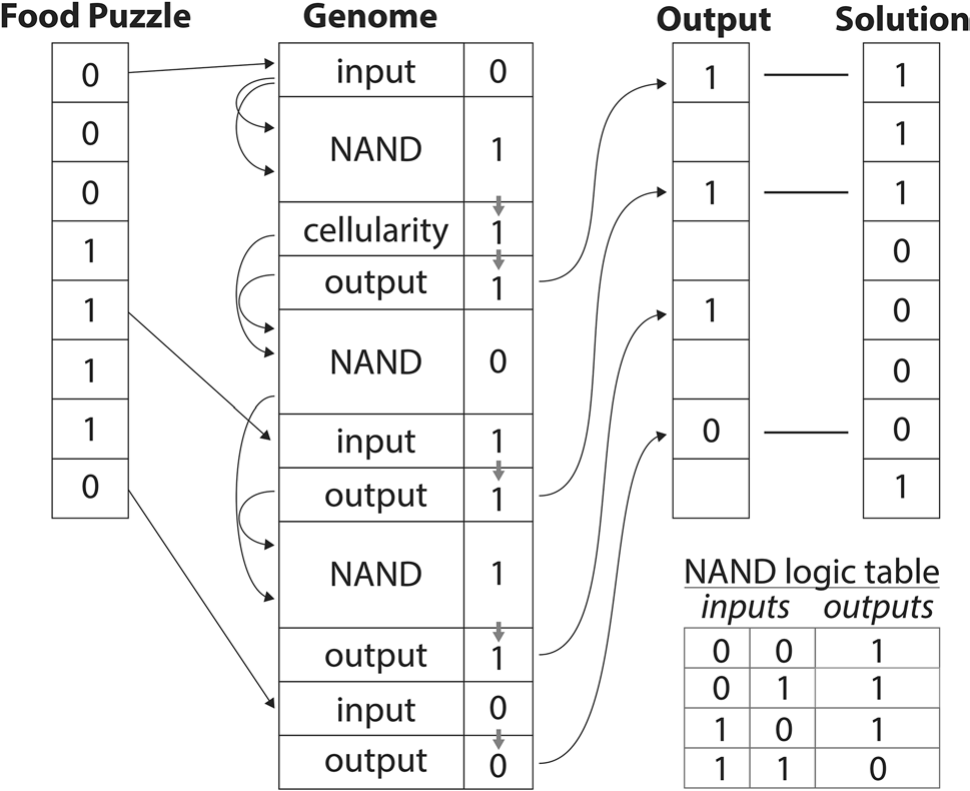
Schematic of a simulated genome and its metabolic processing within the artificial life simulation system. The genome is composed of four types of genes. *Cellularity genes* define the cellular impermeability value of the organism in relationship to a certain function, typically 1–0.5^*n*^, where n = the number of cellularity genes in the genome. Three different *metabolic genes* allow the organism to process food puzzles in order to acquire processing energy. The food puzzles are strings of eight binary digits. The three types of metabolic genes are *input*, *output*, and *NAND*. A read head processes each metabolic gene in the genome in order and associates a binary digit with each gene as it is processed. *Input genes* copy one of the food puzzle digits according to an index number associated with each specific input gene. *NAND genes* contain pointers that take two binary digits associated with any previous genes as input and compute a new binary digit by performing a NAND operation on them. *Output genes* copy the previous binary digit to an output register according to an index number associated with each output gene. The solution to the food puzzle is a string of the opposite binary digits. Processing energy rewards are allocated based on the number of correct matches between the organism’s output register and the food puzzle solution

The artificial life simulation system consists of populations of organisms that are able to evolve increasingly proficient metabolism and impermeable cellular boundaries. Organisms in the simulation can convert food puzzles to processing energy, which is required to run their metabolism and replicate. Organisms replicate automatically if they accumulate a store of processing energy equivalent to four times their genome length. The genome of each organism consists of metabolic and cellularity genes (Fig. 1). Metabolic genes encode a series of pointers and logic gates that allow the organism to solve food puzzles. Cellularity genes simply confer a level of cellular impermeability to the organism defined by a cellularity function, which in most simulations is *cellular impermeability* = 1–0.5^*n*^, where n is the total number of cellularity genes.

Organisms with a low cellular impermeability are leakier insofar as they have a higher likelihood of losing genetic material and processing energy to the environment or gaining genetic material and processing energy from the environment. When an organism loses processing energy to the environment, a random amount of its current energy store is put into an energy parcel and that energy parcel is lost to the environmental pool of processing energy. When processing energy is gained from the environment, an energy parcel is randomly drawn from the environmental energy pool. When an organism loses or gains genetic material, a random sequence of genes is transferred to or from the environment, and corresponding metabolic changes are made to the logic gate network. These random gene transfers are not equivalent to the derived horizontal gene transfer processes observed through, for example, microbial plasmid exchange, and thus are not likely to produce the same kinds of benefits to organisms. A value of 0% cellular impermeability indicates a maximum probability of gene and energy exchange with the environment while a value of 100% indicates that an organism is completely closed to these transfers. This artificial life system is described in further detail in the methods section, below.

Several environmental and genetic parameters are variables that can be set to different values at the start of each simulation. As we were completing the refinement of the simulation code and exploring different combinations of these environmental and genetic parameters, we observed that when there was a very large processing energy payoff for metabolizing food puzzles, however incapable that metabolism was, populations in the simulation would consistently remain at a cellular impermeability value of zero. We suspected that processing energy was becoming so abundant in the environment that non-cellularity was selectively advantageous because it allowed organisms to easily obtain that processing energy from the environment. We subsequently conducted a set of simulations in order to explore the effect of surplus environmental processing energy on the evolution of cellularity.

### The Evolution of Cellularity Depends on Environmental Energy Availability

**Fig. 2:**
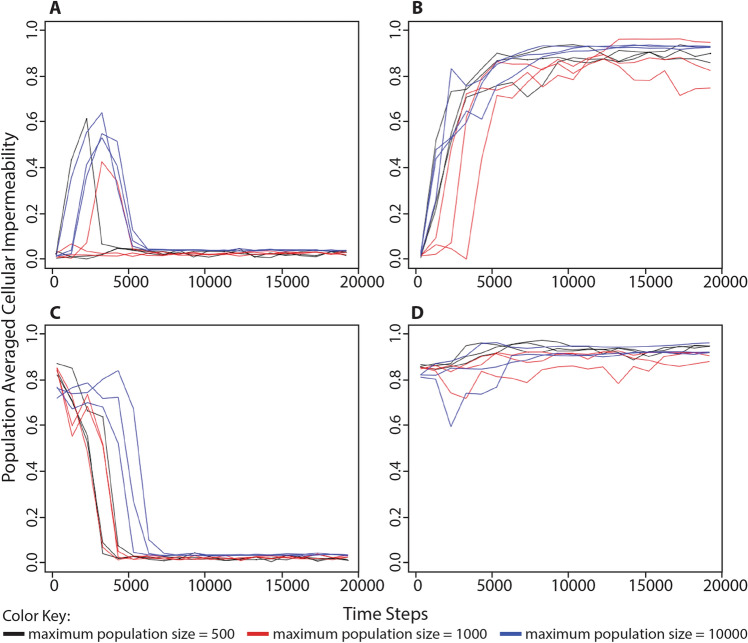
Change in population-averaged cellular impermeability values in different conditions of environmentally available processing energy. A series of simulations were performed to better understand the role of environmentally available energy in the evolution of cellularity. **a** Organisms began each simulation with 0 cellularity genes (i.e., 0% cellular impermeability) in an environment with unlimited food puzzles and processing energy. **b** Organisms began each simulation with 0 cellularity genes (i.e., 0% cellular impermeability) in an environment with unlimited food puzzles, but a limited number of energy parcels in the environment equivalent to 25% the maximum population capacity. **c** Organisms began each simulation with 3 cellularity genes (i.e., 87.5% cellular impermeability) in an environment with unlimited food puzzles and processing energy. **d** Organisms began each simulation with 3 cellularity genes (i.e., 87.5% cellular impermeability) in an environment with unlimited food puzzles, but a limited number of energy parcels in the environment equivalent to 25% the maximum population capacity. Each of the above scenarios was run three times each at three different maximum population sizes: black = 500; red = 1000; blue = 10,000. Simulations were run for 100,000 time steps although only the first 20,000 steps are shown. Cellular impermeability values were recorded every 1000 steps following the first recording at step 250. Taken together, these results demonstrate that environments with limited processing energy select for cellularity, while environments with abundant processing energy select for non-cellularity

We designed a series of simulations to test the hypothesis that the evolution of cellularity is directly influenced by the availability of processing energy in the environment. In the first set of simulations, all organisms began each simulation with 0 cellularity genes (i.e., 0% cellular impermeability). In half of these simulations, the environment was supplied with unlimited processing energy. In this scenario, a low cellular impermeability value was maintained in the population throughout the duration of the simulations (Figs. 2a, S1). In the second set of simulations, the number of energy parcels in the environment was limited to 25% the maximum population capacity. In this scenario, the average cellular impermeability of the population evolved to values between 80% and 100% (Figs. 2b, S2). These results show that low levels of environmental processing energy lead to the evolution of cellularity and suggest that there is an adaptive advantage to cellularity when processing energy is not available within the environment.

The above simulations did not, however, directly demonstrate that high levels of environmental processing energy selects for non-cellularity because the simulations started with organisms already at 0% cellular impermeability. To test whether this sort of environment was actually selecting for non-cellularity, we ran a set of simulation in which organisms began with 3 cellularity genes (i.e., 87.5% cellular impermeability). As predicted, when the environment was supplied with unlimited processing energy, populations evolved average cellular impermeability values very close to 0%, i.e., non-cellularity (Figs. 2c, S3). When the number of energy parcels in the environment was limited to 25% the maximum population capacity, populations maintained high average cellular impermeability values (Figs. 2d, S4).

**Fig. 3:**
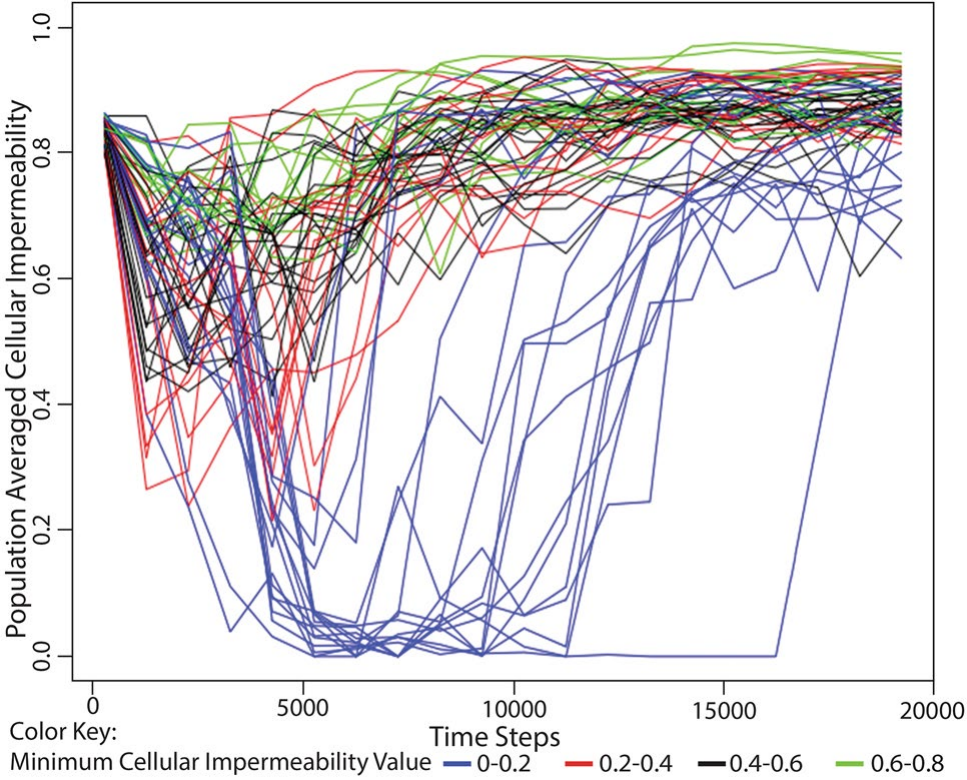
Change in population-averaged cellular impermeability levels in response to changing conditions of environmentally available processing energy. Each of these simulations began with a large pool of processing energy in the environment (10,000 energy parcels with 500 processing energy units, each). The initial environmental energy pool was not replenished and thus diminished over time due to its consumption by organisms. Organisms at the beginning of each simulation had three cellularity genes (i.e., 87.5% cellular impermeability). These simulations were run 100 times each with a maximum population size of 1000. Out of the 100 simulations, 66 had populations that survived to the 100,000th time step while 34 had populations that died out (not shown). Simulations are color-coded by the minimum population-averaged cellular impermeability value: 0–0.2 (blue), 0.2–0.4 (red), 0.4–0.6 (black), and 0.6–0.8 (green). The populations in all simulations showed a temporary decrease in average cellular impermeability in response to environmentally available processing energy and a subsequent increase in average cellular impermeability after that processing energy resource was exhausted. These results demonstrate that the selection pressures favoring non-cellularity in an energy-rich environment and favoring cellularity in an energy-poor environment are reversible and that populations can often survive that reversal

In order to explore whether the populations could adapt to a reversal of selection pressure, we ran simulations in which we provided a burst of environmental processing energy at the beginning of the simulation but did not replenish it afterward. All populations first evolved toward lower levels of cellular impermeability (often reaching a cellular impermeability of 0%), but as the available environmental energy was exhausted, populations began to evolve a higher level of cellular impermeability, eventually reaching values close to the maximum allowed within our digital life environment (Figs. 3, S5, S6). We ran these simulations 100 times in order to test how often populations could survive the reversal of selection pressure. Out of 100 simulations, 66 populations survived this reversal of selection pressure and reached the 100,000th step. This result demonstrates that selection favoring non-cellularity is reversible within the context of our model and occurs as a response to the environment. The results of all of these simulations are presented in Figs. 2 and 3 as population-averaged values, while a more complete view of the changing population over time can be observed in animated gif files available as supplemental files S1–S6.

### Cellularity Coevolves with Metabolism

Our motivating hypothesis, that non-cellularity is selectively advantageous when processing energy is abundant in the environment, implies a corollary hypothesis that cellularity becomes beneficial when organisms have to perform metabolism in order to make their own processing energy from food puzzles. Throughout the simulations described in the previous section, we also measured every organism’s metabolic proficiency. To measure metabolic proficiency, each organism in a simulation is required to solve 1000 randomly generated food puzzles initially and whenever its genome is altered; the average number of correctly solved digits in the food puzzles determines that organism’s metabolic proficiency. An optimal metabolic network has a value of 8 while a random genome will likely have a value between 2 and 4. Metabolic proficiency and cellular impermeability were measured at the same time points across all of the simulations described above.

**Fig. 4:**
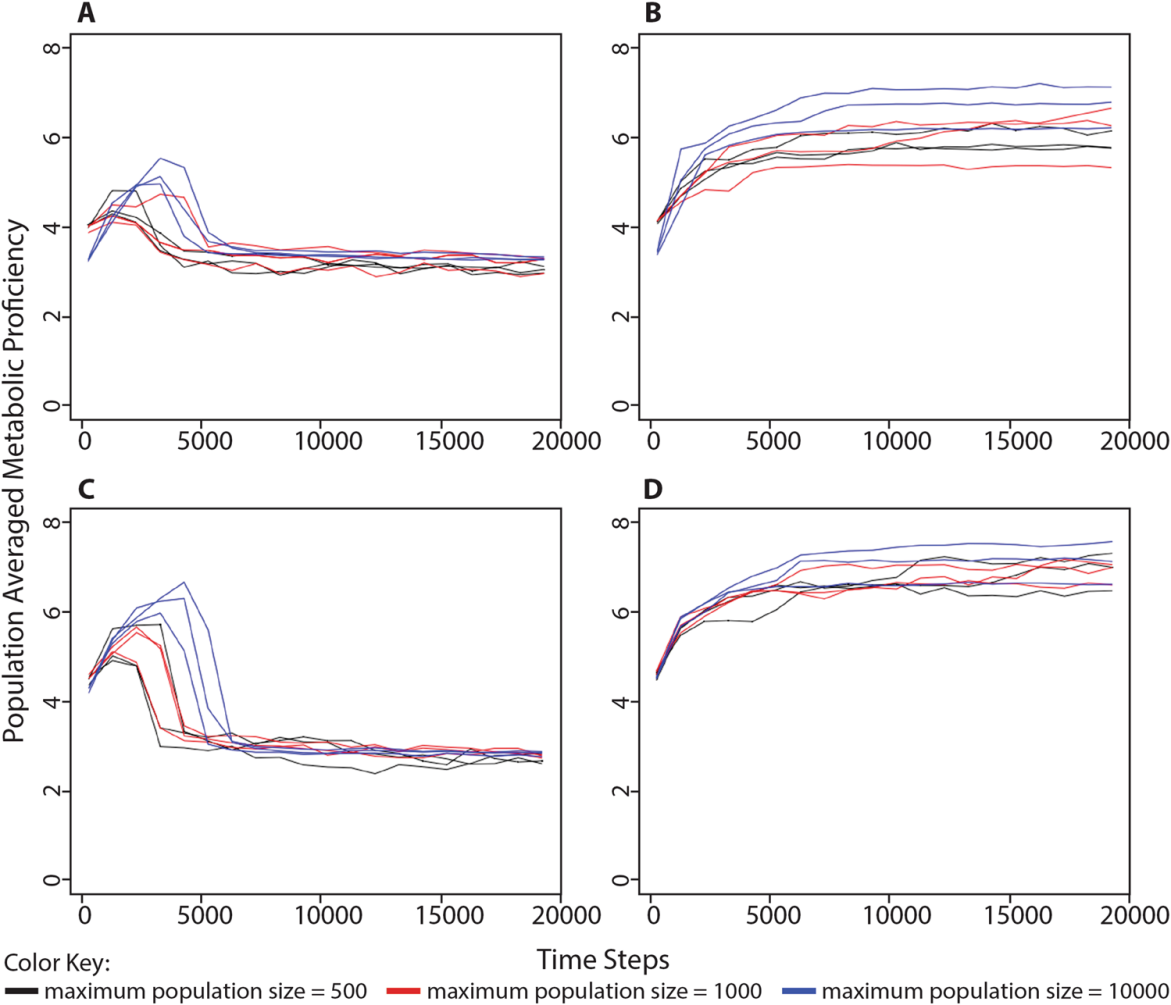
Change in population-averaged metabolic proficiency in different conditions of environmentally available energy. The population-averaged metabolic proficiency was measured alongside the series of simulations described in Fig. 2. **a** Organisms began each simulation with 0 cellularity genes (i.e., 0% cellular impermeability) in an environment with unlimited food puzzles and processing energy. **b** Organisms began each simulation with 0 cellularity genes (i.e., 0% cellular impermeability) in an environment with unlimited food puzzles, but a limited number of energy parcels in the environment, equivalent to 25% the maximum population capacity. **c** Organisms began each simulation with 3 cellularity genes (i.e., 87.5% cellular impermeability) in an environment with unlimited food puzzles and processing energy. **d** Organisms began each simulation with 3 cellularity genes (i.e., 87.5% cellular impermeability) in an environment with unlimited food puzzles, but a limited number of energy parcels in the environment, equivalent to 25% the maximum population capacity. Each of the above scenarios was run three times each at three different maximum population sizes: black = 500; red = 1000; blue = 10,000. Simulations were run for 100,000 time steps although only the first 20,000 steps are shown. Cellular impermeability values were recorded every 1000 steps following the first recording at step 250. Taken together, these results demonstrate that environments with limited environmentally available processing energy select for an increase in metabolic proficiency, while environments with abundant processing energy select for a level of metabolic proficiency equivalent to that of a random genome

In simulations where processing energy was freely available in the environment and where we had previously observed a population-wide selection for non-cellularity, the average metabolic proficiency always settled at a value between 2 and 4 (i.e., the metabolic proficiency of a random genome). This observation held whether the starting cellular impermeability value was 0% (Figs. 4a, S7) or 87.5% (Figs. 4c, S8). In simulations where environmental energy parcels were limited to 25% the maximum population capacity, wherein we had previously observed selection for cellularity, the population-averaged metabolic proficiency consistently increased. This was true whether the starting cellular impermeability value was 0%, where metabolic proficiency settled between 5 and 7 (Figs. 4b, S9) or the starting cellular impermeability value was 87.5%, where metabolic proficiency settled between 6 and 8 (Figs. 4d, S10).

**Fig. 5:**
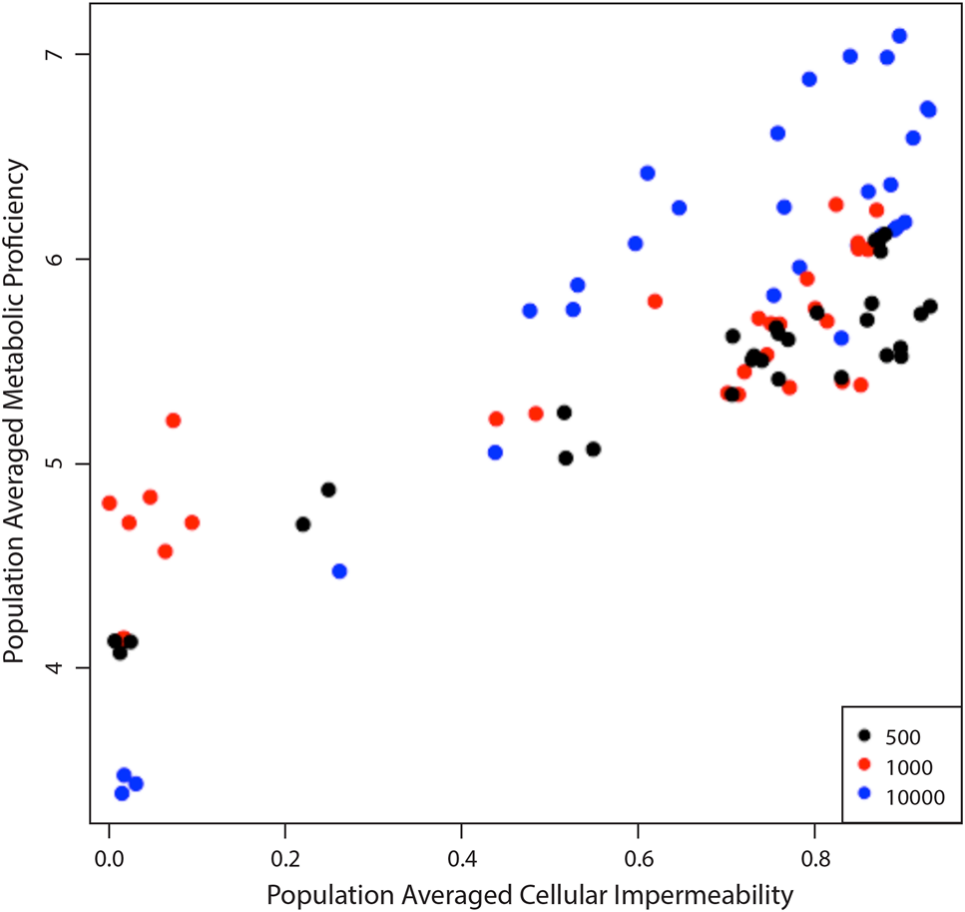
The relationship between cellular impermeability and metabolic proficiency. Results are shown from the simulations in which organisms began with 0 cellularity genes (i.e., 0% cellular impermeability) in an environment with unlimited food puzzles, but a limited number of energy parcels. Cellular impermeability and metabolic proficiency values were recorded every 1000 steps following the first recording at step 250. Color coding refers to the maximum population size of each simulation: black = 500; red = 1000; blue = 10,000. In these simulations, both population-averaged cellularity and populations-averaged metabolic proficiency increased (Figs. 2b and 4b). When shown as a scatterplot, it is clear that cellular impermeability and metabolic proficiency evolve together. Furthermore, the population-averaged values of these traits are very strongly correlated (*r* = 0.84, *p* = 6.07 × 10^–25^), indicating that they are coevolving

These results demonstrate that when populations evolve a high level of cellular impermeability, they also evolve a high level of metabolic proficiency. Between these initial and final values, we also observed that cellular impermeability and metabolic proficiency appeared to increase or decrease alongside one another during the process of evolution. In the simulations where organisms began with a cellular impermeability value of 0% and environmental energy parcels were limited (Figs. 2b and 4b), we tracked the population-averaged values of cellular impermeability and metabolic proficiency as both increased over time (Fig. 5) and found that these values are very strongly correlated (*r* = 0.84, *p* = 6.07 × 10^–25^). These results demonstrate that cellularity and metabolism are coevolving within our simulations. Again, the results of all of these simulations are presented in Figs. 4 and 5 as population-averaged values, while a more complete view of the changing population over time can be observed in animated gif files available as Supplemental Files S7–S10.

### Different Cellularity Functions Produce the Same Results

The earliest forms of cellularity may have been produced endogenously by the organism or may have been imposed by the environment through abiotically produced membrane constituents. To explore the evolutionary consequences of these different scenarios, and to more generally test whether our initial results are robust to changes in the cellularity function, we performed simulations similar to those described above but with different functions for determining an organism’s level of cellular impermeability based on the number of cellularity genes in its genome. The first of these functions confers an organism’s cellular impermeability as equivalent to 0.5^*n*^, where n is the total number of cellularity genes. Here, cellular impermeability is 100% when the organism has 0 cellularity genes and diminishes to 0.5, 0.25, 0.125, etc., as the organism accumulates cellularity genes. We henceforth refer to this function as the reverse cellularity function, because it acts like the reverse of the original cellularity function. The second function is a linear function in which cellularity is equivalent to 0.25*n, where n is the total number of cellularity genes. We henceforth call this function the linear cellularity function.

**Fig. 6:**
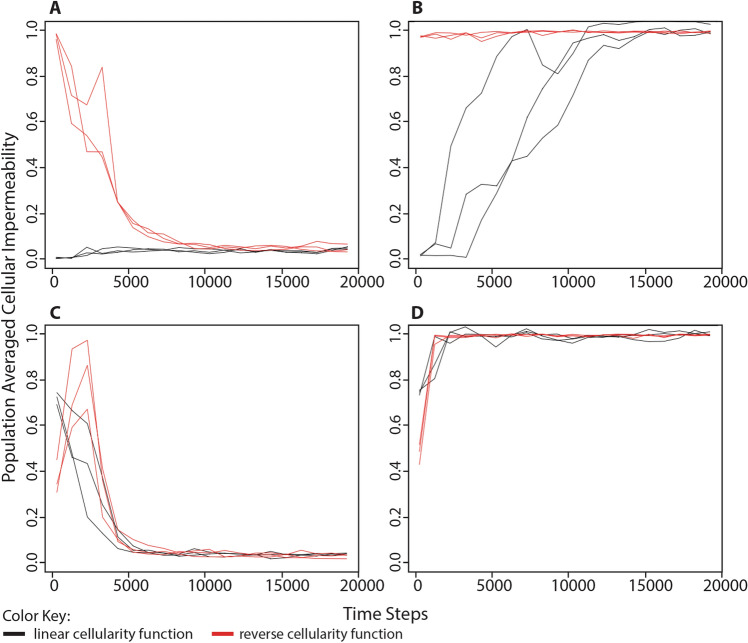
Change in population-averaged cellular impermeability levels in different conditions of environmental energy using alternate cellularity function. The same simulations were performed as shown in Fig. 2 except that the level of cellular impermeability was determined by the number of cellularity genes using either a linear function, cellular impermeability = 0.25*n*, shown in black, or a reverse of the original function, cellular impermeability = 0.5^*n*^, shown in red. **a** Organisms began each simulation with 0 cellularity genes (i.e., 0% cellular impermeability in the linear function or 100% cellular impermeability in the reverse function) in an environment with unlimited food puzzles and processing energy. **b** Organisms began each simulation with 0 cellularity genes in an environment with unlimited food puzzles, but a limited number of energy parcels in the environment equivalent to 25% the maximum population capacity. **c** Organisms began each simulation with 3 cellularity genes (i.e., 75% cellular impermeability in the linear function or 12.5% cellular impermeability in the reverse function) in an environment with unlimited food puzzles and processing energy. **d** Organisms began each simulation with 3 cellularity genes in an environment with unlimited food puzzles, but a limited number of energy parcels in the environment equivalent to 25% the maximum population capacity. These results demonstrate that the previously observed evolution of cellularity in response to the availability of processing energy in the environment is not affected by changes in the way that cellularity is determined by genome content

**Fig. 7:**
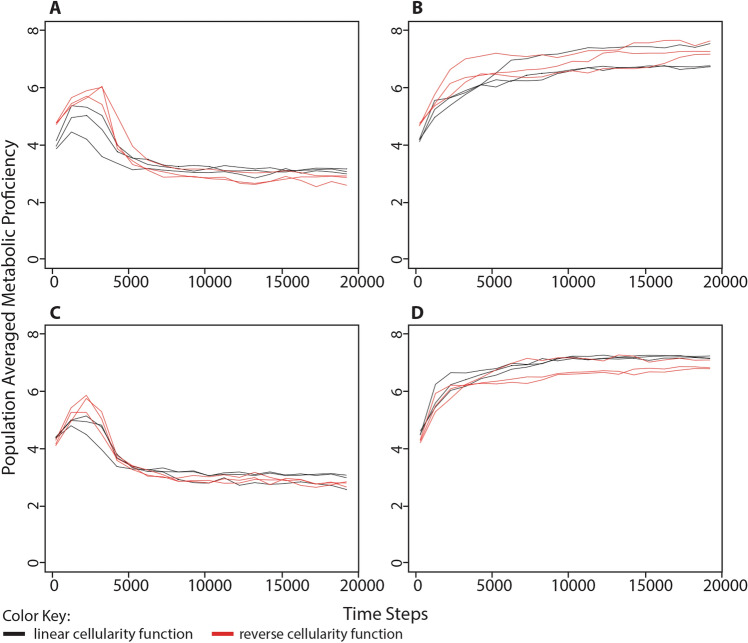
Change in population-averaged metabolic proficiency in different conditions of environmental energy using alternate cellularity function. The population-averaged metabolic proficiency was measured alongside the series of simulations described in Fig. 6, wherein the level of cellular impermeability for each organism was determined by the number of cellularity genes using either a linear function, cellularity = 0.25*n*, shown in black, or a reverse of the original function, cellularity = 0.5^*n*^, shown in red. **a** Organisms began each simulation with 0 cellularity genes (i.e., 0% cellular impermeability in the linear function or 100% cellular impermeability in the reverse function) in an environment with unlimited food puzzles and processing energy. **b** Organisms began each simulation with 0 cellularity genes in an environment with unlimited food puzzles, but a limited number of energy parcels in the environment equivalent to 25% the maximum population capacity. **c** Organisms began each simulation with 3 cellularity genes (i.e., 75% cellular impermeability in the linear function or 12.5% cellular impermeability in the reverse function) in an environment with unlimited food puzzles and environmental energy. **d** Organisms began each simulation with 3 cellularity genes in an environment with unlimited food puzzles, but a limited number of energy parcels in the environment equivalent to 25% the maximum population capacity. These results demonstrate that after substituting very different functions for determining cellular impermeability values, the evolution of metabolism behaves the same as previously observed in simulations using the original cellularity formula (Fig. 4)

Both alternate cellularity functions produce results similar to the original set of simulations. In simulations where processing energy was freely available in the environment, we again observe selection for non-cellularity (Figs. 6a, c). This was true whether organisms started at a low cellular impermeability value, 3 genes for the reverse cellularity function (i.e., 12.5% cellular impermeability) or 0 genes for the linear cellularity function (i.e., 0% cellular impermeability); or started at a high cellular impermeability value, 0 genes for the reverse cellularity function (i.e., 100% cellular impermeability) or 3 genes for the linear cellularity function (i.e., 75% cellular impermeability). In simulations where processing energy was limited in the environment, we again observed selection for high levels of cellular impermeability (Fig. 6b, d). This was also true whether organisms started at low cellular impermeability values or high cellular impermeability values described above. Similarly, under both alternate cellularity functions, we observed selection for low metabolic proficiency when environmental processing energy was unlimited (Fig. 7a, c) and selection for high metabolic proficiency when environmental processing energy was limited (Fig. 7b, d), coinciding with selection for non-cellularity or cellularity, respectively. These results demonstrate that our previous observation regarding the evolution of cellularity and metabolism in response to the amount of environmentally available processing energy was not due to the way in which cellularity genes encode the cellular impermeability phenotype.

### Cellularity-Dependent Food Puzzle Exchange Limits the Evolution of Cellularity and Metabolism

**Fig. 8:**
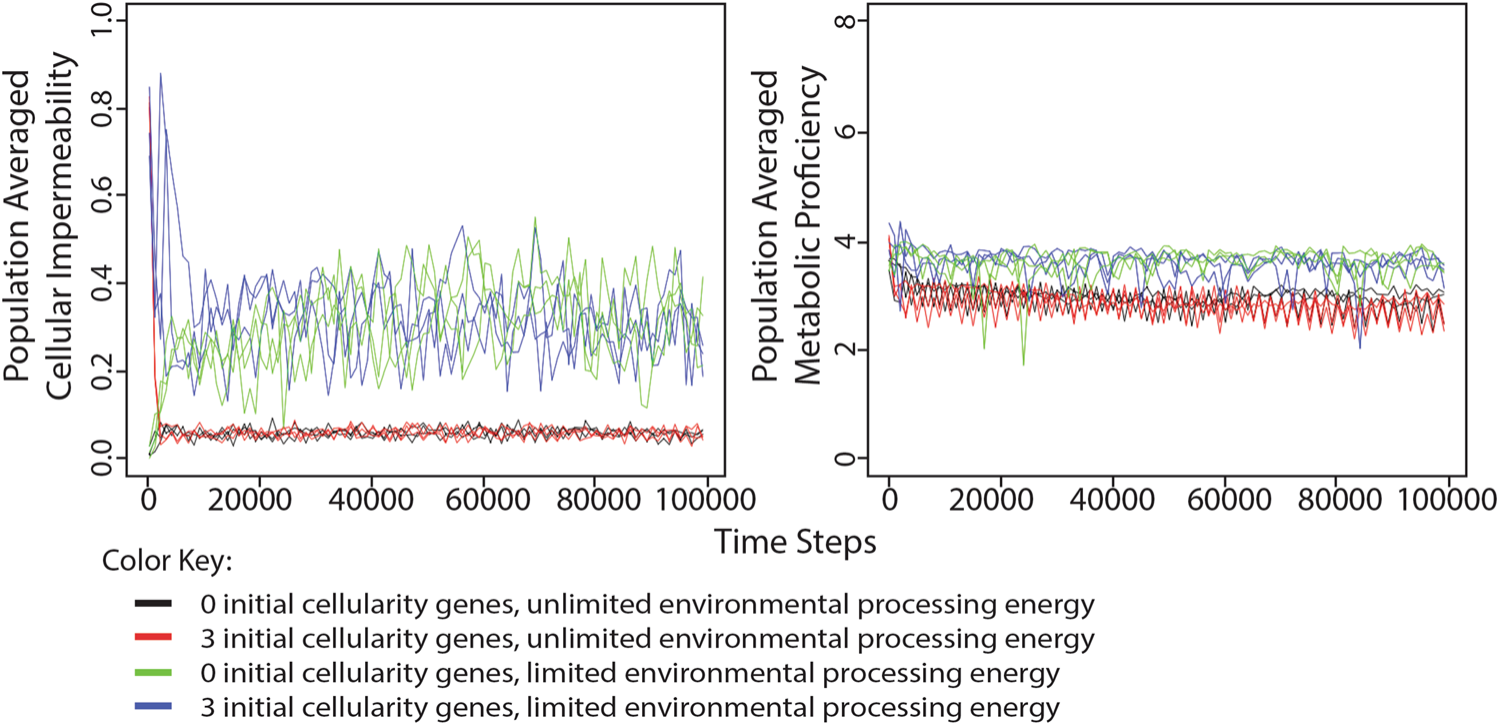
Change in population-averaged cellular impermeability and metabolic proficiency levels in different conditions of environmental processing energy when the acquisition of food puzzles is dependent on cellular impermeability. The simulations were performed in a manner similar to those shown in Figs. 2 and 4 except that the ability of organisms to acquire food puzzles from the environment was dependent on their level of cellular impermeability. Results for both population averaged cellularity (left) and population averaged metabolic proficiency (right) are shown for three repetitions of each of four different simulations: organisms began each simulation with 0 cellularity genes in an environment with unlimited food puzzles and processing energy (black); organisms began each simulation with 3 cellularity genes in an environment with unlimited food puzzles and processing energy (red); organisms began each simulation with 0 cellularity genes in an environment with unlimited food puzzles, but a limited number of processing energy parcels in the environment equivalent to 25% the maximum population capacity (green); or organisms began each simulation with 3 cellularity genes in an environment with unlimited food puzzles, but a limited number of processing energy parcels in the environment equivalent to 25% the maximum population capacity (blue). These results show that when food puzzle acquisition is tied to cellular impermeability, populations do not evolve high levels of cellular impermeability and metabolic proficiency, even under conditions that favored these traits in the original simulations

In our original simulation, the probability of gain or loss of processing energy from or to the environment was dependent on the cellular impermeability value of the organism, but the cellular impermeability value had no effect on food puzzle acquisition. That is to say, even an organism with near 100% cellular impermeability could always acquire food from the environment. To test the effects of cellularity-dependent food puzzle exchange, we designed simulations in which the probability of food puzzle acquisition was dependent on the level of cellular impermeability in the same manner as transfers of processing energy and genetic material (Fig. 8).

As we observed in the original simulations, low levels of cellular impermeability evolved when processing energy was freely available in the environment. This was true whether the starting value of cellular impermeability was low (0%) or high (87.5%). The selection for low cellular impermeability coincided with a selection for low levels of metabolic proficiency just as it had in the original simulations. But the results of these simulations differed from those of the original simulations when processing energy in the environment was limited. Here, the population averaged level of cellular impermeability hovered around 0.3 whether the starting value of cellular impermeability was low (0%) or high (87.5%) and the metabolic proficiency value was only slightly higher compared to the simulations in which processing energy was freely available. These results suggest that cellularity-dependent food puzzle exchange creates a selection pressure that favors non-cellularity because organisms with lower cellular impermeability have a higher probability of accessing food in the environment. The resulting evolution toward middling levels of cellular impermeability is likely the result of a balance between this selection pressure and the selection favoring high levels of cellular impermeability that was observed in previous simulations.

### Specific Causes of Selection Pressures Favoring or Opposing Cellularity

Having established a very strong correlation between the evolution of cellularity and the evolution of metabolism, we designed several simulations in order to better understand the underlying causes of the selection pressures that led to cellularity or non-cellularity. Cellularity may be selectively advantageous when processing energy in the environment is scarce because it benefits organisms to retain the products of their own metabolism. Alternatively, cellularity may be selectively advantageous when processing energy in the environment is scarce because organisms cannot maintain a high metabolic proficiency when random gene transfers are common and act as an additional source of mutation. Similarly, selection may favor non-cellularity when processing energy in the environment is plentiful because it provides access to that processing energy, or because cellularity genes increase the size of the genome and thereby increase the cost of genome processing. To address these alternate explanations, we repeated the previous simulations in which organisms either started with 0% or 87.5% cellular impermeability and the environment was either seeded with unlimited processing energy or with a limited number of energy parcels equivalent to 25% the maximum population capacity. However, in these simulations, we manipulated the consequences of cellularity such that the probability of random gene transfers or energy transfers was set to a constant value of either 0% or 100% rather than being determined by the level of cellular impermeability.

**Fig. 9:**
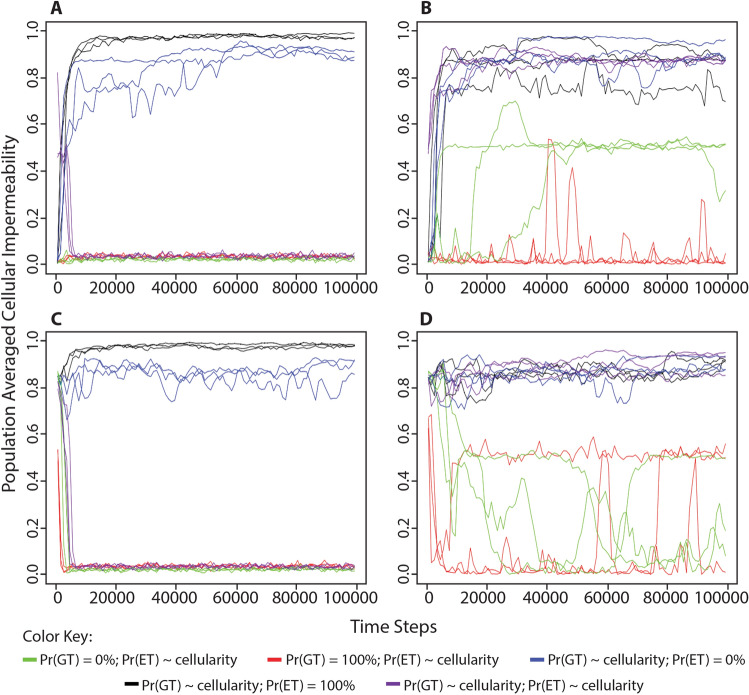
Change in population-averaged cellular impermeability levels in different conditions of environmental processing energy when either random gene transfer or energy transfer is no longer a consequence of cellularity. We performed the simulations described in Figs. 2 and 4. **a** Organisms began each simulation with 0 cellularity genes (i.e., 0% cellular impermeability) in an environment with unlimited food puzzles and processing energy. **b** Organisms began each simulation with 0 cellularity genes (i.e., 0% cellular impermeability) in an environment with unlimited food puzzles, but a limited number of processing energy parcels in the environment, equivalent to 25% the maximum population capacity. **c** Organisms began each simulation with 3 cellularity genes (i.e., 87.5% cellular impermeability) in an environment with unlimited food puzzles and processing energy. **d** Organisms began each simulation with 3 cellularity genes (i.e., 87.5% cellular impermeability) in an environment with unlimited food puzzles, but a limited number of processing energy parcels in the environment, equivalent to 25% the maximum population capacity. The consequences of cellularity are manipulated as such: Green = probability of gene transfer is set to 0%; Red = probability of gene transfer is set to 100%; Blue = probability of processing energy transfer is set to 0%; Black = probability of processing energy transfer is set to 100%; Purple = no manipulation. All simulations were performed with a maximum population size of 1000

**Fig. 10:**
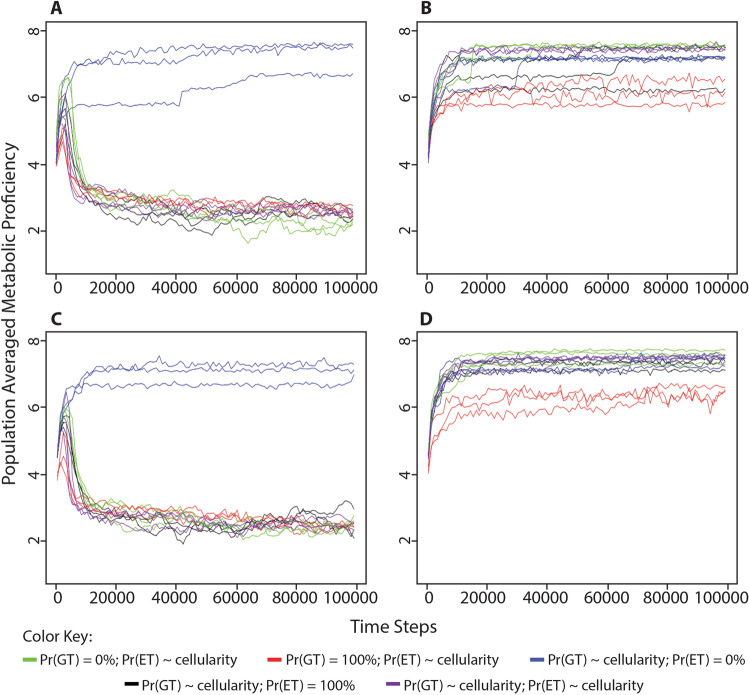
Change in population-averaged levels of metabolic proficiency in different conditions of environmental processing energy when either random gene transfer or energy transfer is no longer a consequence of cellularity. We performed the simulations described in Figs. 2 and 4. **a** Organisms began each simulation with 0 cellularity genes (i.e., 0% cellular impermeability) in an environment with unlimited food puzzles and processing energy. **b** Organisms began each simulation with 0 cellularity genes (i.e., 0% cellular impermeability) in an environment with unlimited food puzzles, but a limited number of processing energy parcels in the environment, equivalent to 25% the maximum population capacity. **c** Organisms began each simulation with 3 cellularity genes (i.e., 87.5% cellular impermeability) in an environment with unlimited food puzzles and processing energy. **d** Organisms began each simulation with 3 cellularity genes (i.e., 87.5% cellular impermeability) in an environment with unlimited food puzzles, but a limited number of processing energy parcels in the environment, equivalent to 25% the maximum population capacity. The consequences of cellularity are manipulated as such: Green = probability of gene transfer is set to 0%; Red = probability of gene transfer is set to 100%; Blue = probability of energy transfer is set to 0%; Black = probability of energy transfer is set to 100%; Purple = no manipulation. All simulations were performed with a maximum population size of 1000

The simulations in which random gene transfer was held constant (either at 0% or 100%) demonstrated that cellularity is selectively advantageous because it contributes to genetic fidelity. In all simulations in which processing energy was freely available in the environment, non-cellularity was selected (Fig. 9a, c; green, red) just as it was when the probability of random gene transfer was determined by the level of cellular impermeability (Fig. 2a, c). However, when processing energy in the environment was limited, the level of cellular impermeability remained lower (Fig. 9b, d; green, red), which differed from previous simulations in which the probability of random gene transfer was determined by the level of cellular impermeability (Fig. 2b, d). Furthermore, when random gene transfer was held constant, cellular impermeability did not consistently settle at a particular value even after 100,000 time steps. Even though high levels of cellular impermeability did not evolve when random gene transfer was held constant, the population-averaged metabolic proficiency did increase over time (Fig. 10b, d; green, red). Taken together, these results indicate that while non-cellularity was selectively advantageous when processing energy was abundant because it allowed organisms to access that energy, high cellular impermeability was selectively advantageous when processing energy was scarce, at least in part, because it improved genetic fidelity and allowed organisms to evolve genomes that could perform metabolism.

The simulations in which energy transfer was held constant (either at 0% or 100%) confirmed that non-cellularity was selectively advantageous when energy in the environment was unlimited because it allows organisms to access that environmental energy. When energy was available in the environment and the probability of energy transfer was set to a constant value of 0%, populations evolved both a high level of cellular impermeability (Fig. 9a, c; blue) and a high level of metabolic proficiency (Fig. 10a, c; blue), just as they did when the probability of random energy transfer was determined by the level of cellular impermeability and environmental processing energy was limited (Figs. 2b, d; 4b, d). However, when energy transfer was set to a constant value of 100%, the results were less straightforward. Here, populations evolved toward high levels of cellular impermeability (Fig. 9a, c; black) and low levels of metabolic proficiency (Fig. 10a, c black).

It is unlikely that cellularity provided a selective advantage by protecting organisms from random gene transfers in this scenario because populations also evolved low levels of metabolic proficiency and therefore should be insensitive to genomic disruptions. More likely, selection for low cellular impermeability was relaxed because energy transfer remained at the maximum level regardless of the level of cellular impermeability. It is possible that under this scenario, cellularity genes were allowed to accumulate by random chance, leading ultimately to a relatively high level of cellularity. It is already known that genetic drift alone can explain a so-called increase trend when the founding character value is low (Stanley 1973; Gould 1988). Under this hypothesis, the distribution of traits would expand toward progressively higher values under random mutation because the founding character value is bounded on the low end.

**Fig. 11:**
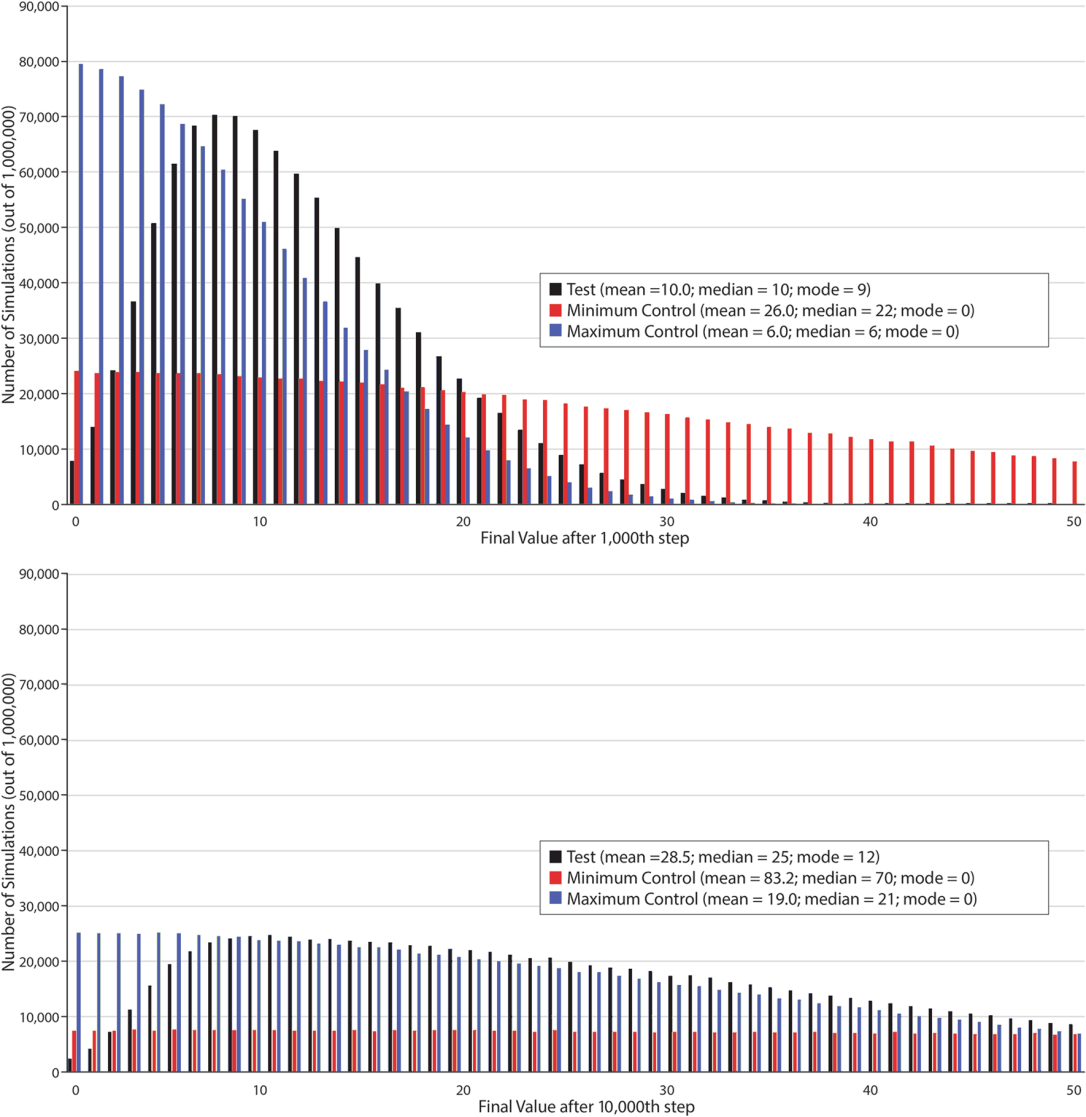
A generalized simulation demonstrating the effect of genetic drift over a trait that determines mutation rates. Because increased cellular impermeability leads to a lower mutation rate, we proposed that genetic drift would favor increased cellular permeability in the absence of any other selection pressures (see Fig. 9, black line). The test simulation mimics the effects in cellularity genes as an evolvable “fidelity value” (probability of mutation = 0.5^*x*^, where x is the fidelity value). An additional mutation step mimics the probability of insertion or deletion mutations in the original model, which are independent of cellularity. Controls mimic the mutation rates of populations with a constant cellular impermeability value of 100% (“Minimum Control”) or 0% (“Maximum Control”). Simulations were run 10^6^ times for each case and fidelity values were recorded at the 1000th step (top) or the 10,000th step (bottom). These simulations show an important effect of genetic drift occurring over a character that affects mutation rate. Specifically, the distribution appears to preclude low values, unlike both controls wherein the mode value is 0. As such, relatively high levels of cellular impermeability are nearly certain to evolve within our model in the absence of any selection pressures

We find, however, that this drift toward high levels of cellular impermeability is further enhanced by the effect of cellular impermeability on mutation rate. High levels of cellular impermeability decrease the likelihood of further mutations. As such, lineages with higher levels of cellular impermeability may be more likely to maintain those higher levels of cellular impermeability than to drift back to lower levels of cellular impermeability because they are less likely to mutate at all. To test this hypothesis, we designed a naïve simulation in which a genetic fidelity trait is allowed to drift randomly, but the higher the level of genetic fidelity, the slower the drift. These simulations used the same statistical properties as the original simulations, but did not include other elements of the original simulations such as genomes, metabolism, replication, and death. In these simulations, when a trait value is linked to mutation rate, the distribution of trait values after genetic drift is altered such that lower values are increasingly precluded over time (Fig. 11). This result suggests that cellularity could have evolved in the absence of any selection pressures simply because it decreases mutation rates and reduces genetic drift.

**Fig. 12:**
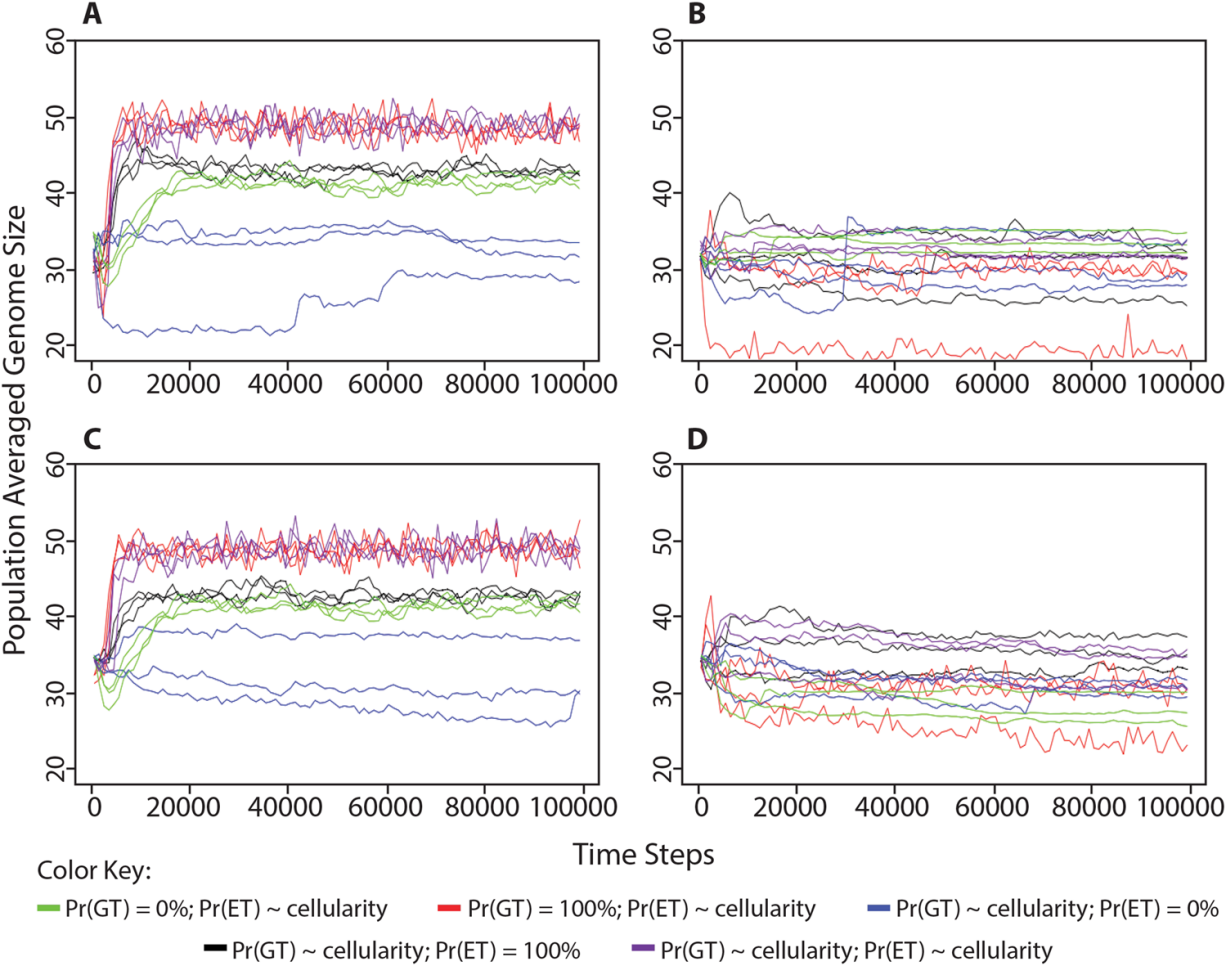
Change in population-averaged genome lengths in different conditions of environmental processing energy when either random gene transfer or energy transfer is no longer a consequence of cellularity. We performed the simulations described in Figs. 2 and 4. **a** Organisms began each simulation with 0 cellularity genes (i.e., 0% cellular impermeability) in an environment with unlimited food puzzles and processing energy. **b** Organisms began each simulation with 0 cellularity genes (i.e., 0% cellular impermeability) in an environment with unlimited food puzzles, but a limited number of processing energy parcels in the environment, equivalent to 25% the maximum population capacity. **c** Organisms began each simulation with 3 cellularity genes (i.e., 87.5% cellular impermeability) in an environment with unlimited food puzzles and processing energy. **d** Organisms began each simulation with 3 cellularity genes (i.e., 87.5% cellular impermeability) in an environment with unlimited food puzzles, but a limited number of processing energy parcels in the environment, equivalent to 25% the maximum population capacity. The consequences of cellularity are manipulated as such: Green = probability of gene transfer is set to 0%; Red = probability of gene transfer is set to 100%; Blue = probability of energy transfer is set to 0%; Black = probability of energy transfer is set to 100%; Purple = no manipulation. All simulations were performed with a maximum population size of 1000

Under all of the above simulations, we also measured genome lengths along with levels of cellular impermeability and metabolic proficiency (Fig. 12). We did so in order to determine whether selection was acting on genome size. Interestingly, when processing energy in the environment was limited (Fig. 12b, d), the population-averaged genome length was always relatively low (~ 20–30 genes). When processing energy in the environment was abundant (Fig. 12a, c) population-averaged genome length was usually relatively high (40–50 genes). An exception to this trend was that the population-averaged genome length was also always relatively low, regardless of the availability of processing energy in the environment, when the probability of energy transfer was not a consequence of cellularity, but was instead set to a constant value of 0%. Thus, environments with limited processing energy led to the evolution of smaller genomes, likely because the energy required to process those genomes would have been harder to acquire.

While the initial observation that cellularity and metabolism coevolve is straightforward, these simulations demonstrate that the underlying causes are complex. When processing energy was abundant in the environment, selection favored non-cellular organisms because it allowed them to access that environmental energy. Aside from maintaining low levels of cellular impermeability, there was no selection pressure on the genome itself, either to produce a proficient metabolism or to maintain a low genome size. When processing energy in the environment was limited and organisms had to produce their own energy from metabolism, cellularity was selectively advantageous because it allowed organisms to maintain a genome that could perform metabolism, not because it allowed organisms to retain the energy that they created from that metabolism. Furthermore, organisms evolved smaller genomes under these conditions despite the need to produce processing energy through genomically encoded metabolism. Thus, scenarios that favored organisms with a proficient metabolism also favored organisms that produced that metabolism efficiently by way of a relatively short genome. Finally, in the absence of any selection pressure, cellularity could evolve by a directional genetic drift tending toward higher values of cellular impermeability that correspond with lower mutation rates.

## Discussion

The evolution of cellularity was one of the most important transitions in evolutionary history. The results presented above contribute to our understanding of this important stage in evolutionary history by demonstrating that cellularity and metabolism coevolve in response to environmental change. Specifically, we found that environments rich in processing energy selected for low cellular impermeability and low metabolic proficiency while environments poor in processing energy selected for high cellular impermeability and high metabolic proficiency and that between these two extremes, the population-averaged values of cellular impermeability and metabolic proficiency were very strongly correlated.

By setting random gene transfer and energy transfer to constant values, we were able to ascribe causes to these evolutionary trends. When processing energy was freely available in the environment, non-cellularity was advantageous because it allowed organisms to easily access that energy. Organisms did not need to produce their own processing energy from food puzzles and metabolic proficiency did not evolve beyond the value range equivalent to a random genome. When processing energy was not abundant in the environment, organisms had to produce their own energy from food in order to survive and replicate. This scenario created selection pressure for high levels of metabolic proficiency and also for high levels of cellular impermeability, which was essential for maintaining the genetic fidelity required to sustain a genome that is capable of producing an effective metabolism. However, when no obvious selection pressures were present, cellularity appeared to evolve by a directional genetic drift that favored relatively high levels of cellular impermeability because they caused a decrease in the mutation rate and thereby the rate of further genetic drift.

We find that the evolutionary relationship between cellularity and metabolism is not dependent on the cellularity function, itself, as we observed similar dynamics when the original cellularity function was replaced with a linear or (more surprisingly) a reverse cellularity function. The latter cellularity function has especially important implications for the origin and early evolution of life because it mimics a scenario in which cellularity is imposed by the environment, rather than endogenously produced by the organisms. In this scenario, organisms had to acquire reverse cellularity genes to diminish the cellular impermeability value below 100%. We found that in simulations that favor non-cellularity, i.e., when processing energy in the environment was unlimited, organisms did indeed accumulate these reverse cellularity genes and thereby acquire low levels of cellular impermeability. In a potential origin of life scenario that included lipid membranes, the same selection pressures could favor the disruption of these membranes, or, alternatively, could favor the evolution of non-specific membrane pores, selective membrane channels, or even transporters, assuming that the genetic system had evolved a sufficient level of complexity to produce such gene products.

The results of our simulations are, on the other hand, sensitive to whether food puzzles can be acquired by the organism regardless of its cellularity value. In our original simulations, organisms were just as likely to acquire food if their cellular impermeability value was 100% as they were if their cellular impermeability value was 0%. This dynamic is similar to some autotrophic metabolisms, e.g., photosynthesis and some forms of chemosynthesis such as methanogenesis, in which the metabolic inputs (i.e., light, CO_2_, H_2_O, or H_2_) can easily traverse cell membranes even though the metabolic outputs cannot. Other forms of metabolism, especially heterotrophic metabolisms, require the active transport of nutrients that cannot freely pass through cell membranes. We found that when we set the probability of food puzzle acquisition as a function of the cellular impermeability value, the population-averaged value of cellular impermeability increased, but was never higher than around 50%. These results suggest that cellularity would not have been selectively advantageous in heterotrophic organism prior to the advent of membrane transporters that could have brought large organic nutrients into the interior of the cell.

Cellularity as we have modeled it in our artificial life simulations is certainly a simplified abstraction. A broad range of molecular functions is required to establish the kind of cellularity we see today, including the metabolic synthesis of phospholipids and other membrane constituents, the molecular system for targeting proteins and other biomolecules into and across the cell membrane, and the ability to faithfully replicate a cell into two similar if not identical cells. Here we simply model cellularity by the accumulation of general cellularity genes. However, in our model, the genomic cost of maintaining cellularity is slightly higher than in modern organisms. In an initialized genome in which organisms have a cellular impermeability value of 87.5%, the cellularity genes make up 11.1% of the genome and the ratio of cellularity genes to metabolic genes is 1:8. In *Escherichia coli*, genes that encode functions related to cellularity account for 4.1% of the proteins in the genome and the ratio of cellularity genes to metabolic genes is 1:23.6 (PANTHER DB version 14.1; Thomas et al. 2003; Mi et al. 2013). Therefore, even though our model for genetically encoding cellularity is simple in comparison to real organisms, the genomic cost of maintaining cellularity is actually higher.

The metabolism modeled by our artificial life environment is also a simplified abstraction. Metabolism as we have defined it represents a genome’s ability to solve a problem and therein convert food puzzles to processing energy. The food puzzles represent a nutrient available in the environment and the processing energy represents any product that is synthesized from that nutrient and is essential for the organism to survive and reproduce. As such, our artificial life simulation can incorporate a range of hypotheses about the origin of life. Processing energy in the simulation could represent charged nucleotides in an RNA world, multiple constituents of an autocatalytic network, etc. As such, our results have important consequences for the origin of life regardless of how it specifically occurred.

Most origin-of-life hypotheses require, either explicitly or implicitly, a rich geochemical environment to support the chemical or genetic replicators that preceded *bona fide* organisms (e.g., Rode et al. 1999; Parnell 2004; Martin and Russell 2007; Johnson et al. 2008; Stüeken et al. 2013). The first metabolic pathways are thought to have evolved in order to synthesize important compounds that had previously been, but were no longer being, produced by the rich geochemical setting of life’s origin (Horowitz 1945; Lazcano and Miller 1999; Yamada and Bork 2009). Our results confirm the hypothesis (Szathmáry 2007) that cellularity also emerged during this stage in evolution. As important compounds became scarce in the environment and a capable metabolism evolved to produce those compounds biosynthetically, cellular organization very likely coevolved along with that early metabolism. This cellular organization served to protect proto-organisms from the genomic disruptions of random gene losses and gains. In this way, our results are consistent with previous modeling studies that show that cellular organization may have benefitted early replicators by mitigating the effects of parasites (e.g., Takeuchi and Hogeweg 2009; Matsumura et al. 2016; Shah et al. 2019). In other words, both our results and the results of these previous studies suggest that cellularity was selectively advantageous because it increased genetic fidelity. This increased genetic fidelity, in turn, may have further facilitated the evolution of both metabolic and cellular systems in an evolutionary feedback loop.

Our results also suggest that the emergence of cellularity is more complicated than the basic assumption that life originated as non-cellular and then subsequently evolved cellularity once it had become sufficiently complex. Our simulations show that the kind of rich geochemical environment in which the origin of life likely took place would have maintained life as non-cellular because selection would have favored the replicators that could most easily access important compounds in the environment. Even in a scenario where cellularity was imposed by the environment, not produced endogenously by the organism, our results show that selection favored non-cellularity. This observation does not rule out membrane compartmentalization during the origin of life, but suggests that some additional mechanism for concentrating nutrients within protocells would be required (e.g., Damer and Deamer 2015). Indeed, we find that a selection pressure favoring increased genome stability through cellularity is still present in simulations with rich environments, but that this selection pressure is outweighed by selection favoring non-cellularity as a means of accessing environmental energy.

Taken together, our results indicate that true cellularity, as opposed to transient protocells, likely evolved only after a change in the setting of life’s origin. This reduction in available nutrients may have been due to their depletion in the environment as life multiplied, or a change in the underlying geochemistry that had previously produced those nutrients. As some hypotheses about the origin of metabolic pathways suggest, this environment could have been abundant enough to sustain life during its origin, but depleted soon after, necessitating the evolution of metabolism (Horowitz 1945; Lazcano and Miller 1999; Yamada and Bork 2009). Our results suggest that the evolution of cellular organization likely occurred during this metabolic transition as well. This transition toward cellular organization would have subsequently promoted linear descent, i.e., the transfer of genetic information from parent to offspring, as the principal mechanism of gene transfer. As such, the evolution of cellular organization may have been a prerequisite for the taxonomic diversification (Woese 1998) and the ecological dispersal (Cantine and Fournier 2018) that, together, have shaped the evolution of the biosphere.

## Methods

### Simulation Overview

The artificial life simulation system described above consists of populations of organisms that require processing energy to survive and reproduce, which can be obtained by metabolizing food puzzles available in the environment. Organisms can replicate when excess processing energy is accumulated. The genome of each organism consists of separate metabolic and cellularity genes. Metabolic genes are encoded as a series of pointers and logic gates that allow them to solve food puzzles. Cellularity genes simply confer the overall level of cellular impermeability of the organism as 1–0.5^*n*^, where n is the total number of cellularity genes. Each genome is subjected to a predefined probability of insertions, deletions, and pointer mutations. At each time step, organisms proceed a single step through their genome and consume one unit of processing energy. All mutations, random gene transfers, processing energy transfers, and tests of metabolic proficiency happen at set intervals of 100 time steps. Organisms may be born or die at any given time step. The simulation is built with no spatial component, so organisms are equally likely to gain food, available processing energy, or genes from the environment without any relative positioning of components within the ecosystem. The software we developed to perform these artificial life simulations is available at https://github.com/GoldmanLab/Simmerpop.

#### Food puzzles

A food puzzle consists of a randomly generated eight-bit input register and a solution register. An organism can solve the food puzzle using its metabolic genes in order to obtain processing energy (see below). The solution to each food puzzle is a Boolean NOT operation at every index of the input register. For example, if the food particle is [1,0,1,0,1,1,0,0], its solution is [0,1,0,1,0,0,1,1].

#### Metabolism

The metabolic genes comprise a set of instructions that execute Boolean logic operations to solve the food puzzles. The use of Boolean operators as genes was inspired by the Avida software platform (Lenski et al. 2003; Ofria et al. 2004). Each organism has a genome of variable length and an eight-bit output register. A read head denotes the current instruction being executed. When an organism receives a food puzzle, it starts to execute at the beginning of its genome and progresses linearly one step at a time until the read head reaches the end of the genome. Once finished, the output register is compared against the solution register of the food. The processing energy reward is calculated based on (*n*^3^−*x* × 0.1), where n is the number of correct bits in the output and x is a reduction factor. This reduction factor dynamically adjusts the processing energy reward from food puzzles based on the population size. The reduction factor will increment by one every time step that the population size exceeds 85% of the population capacity. Consequently, the processing energy reward from food puzzles will decrease to increase competition in the population. On the other hand, when the population size drops below 25% of the population capacity, the factor will decrement by one every time step to promote survival by providing more processing energy per solved food puzzle.

There are three different instruction types within the metabolic component of an organism’s genome, which taken together create a Boolean logic gate network (see Fig. 1): (1) An input gene has a pointer referring to one of the eight indices of the food’s input register. When an input instruction is executed, it evaluates to the value referred to by its pointer. (2) An output gene has a pointer referring to the preceding instruction and a pointer referring to one of the eight indices of the organism’s output register. When an output instruction is executed, it evaluates to the same value as the previous instruction, and also places that value in the output register. (3) A NAND gene has 2 pointers referring to certain preceding instructions. When a NAND instruction is executed, it evaluates to the logical NAND (or NOT-AND) of the values of the previous instructions indicated by the pointers and the result is stored in the instruction’s memory slot. Cellularity genes are also interspersed throughout the genome. A cellularity gene has a pointer referring to the preceding instruction so that NAND genes pointing to a cellularity gene receive a value.

To test metabolic proficiency, the organism is required to solve 1000 randomly generated food puzzles at inception, and after any genome mutation; the average accuracy of the solution determines the organism’s metabolic proficiency value. An optimal metabolic strategy will have a value of eight; a random genome will likely have a value between two and four.

#### Cellularity

An organism’s overall level of cellular impermeability is represented by a value between 0 and 1. Except when otherwise noted, the cellular impermeability value is calculated from the number of cellularity genes as 1–0.5^*n*^, where n is the number of cellularity genes. Organisms with a low cellular impermeability have a higher likelihood of losing/gaining genes and processing energy. A value of 0% indicates a complete openness to gene and energy exchange with the environment while a value of 100% indicates that an organism is completely closed to these transfers. In the case of the cellularity-dependent food puzzle transfer simulations, food transfer was tied to cellularity in the same way as gene and energy transfer. These random transfers occur at intervals of 100 time steps.

#### Processing Energy and Replication

Organisms begin each simulation with an initial stock of processing energy equivalent to twice the length of their genome. Organisms can gain processing energy in two ways, by solving food puzzles or by receiving energy parcels from the environment when cellular impermeability is low (see above). However, when cellular impermeability is low, the organism is equally likely to lose processing energy to the environment. All organisms use one unit of processing energy at every time step of a simulation.

An organism replicates once it accumulates a store of processing energy equivalent to four times its genome length. Thus, after the start of the simulation, every new organism begins with half of the processing energy stock of its parent at the time of replication. We tested the effects of a genome-dependent replication cost by imposing an additional energy cost for replication that was equivalent to two times the genome length and not passed down to offspring, but found that this additional replication cost did not alter the results of our simulations (Figure S11).

#### Mutations

Organisms can undergo four different kinds of mutations, insertion mutations, deletion mutations, pointer reassignments, and random gene transfers. Mutations occur at intervals of 100 time steps and each kind of mutation can be assigned a different probability before running the simulation. After an initial exploration of the parameter space, we determined that 0.005 per site was a reasonable mutation probability for insertions, deletions, and pointer reassignments for the purposes of this study.

Random gene transfers occur when the cellular impermeability value is less than 100%. An organism can lose a segment of its genome as small as one instruction or as large as its entire genome. Genes that are lost are added to the free gene pool in the environment. When a gene loss event occurs, the genome is split up into random sections, one of which is selected at random to be lost and added to the free gene pool. The remaining fragments are concatenated back together maintaining their order. When a gene insertion occurs, the genome is broken up in an identical fashion and a new gene from the common gene pool is randomly inserted at one of the break points before the genome is concatenated back together. The likelihood that the sequence will break at any given position is dictated by a break weight probability of 0.1. The probability that a gene transfer happens in a particular organism at a particular time is inversely proportional to the organism’s cellular impermeability value.

#### Structure of a Simulation

At the beginning of every simulation, organisms are given a starting genome and an allocation of energy equivalent to two times the length of their genome. The starting genome consists of eight input instructions, followed by sixteen NAND instructions, and eight output instructions, as well as either zero or three cellularity genes depending on the simulation. This is a hybrid randomized/designed genome: the designed component is in the input and output instructions, which represent an optimal configuration with no redundancy. However, each organism’s starting genome represents a random Boolean logic gate network because the pointers associated with the NAND instructions are randomly assigned.

During the course of a simulation, if an organism does not have a food puzzle, it attempts to acquire one. In all simulations except those presented in Fig. 8, the environmental pool was treated as effectively infinite, so that organisms always received a new food puzzle if needed. If an organism has reached the end of its genome, it calculates the appropriate processing energy reward based on its output solution and gains that amount of processing energy. When an organism’s processing energy stock is four times its genome length, it can divide into two offspring organisms. These offspring organisms’ genomes are identical to that of the parent and they each receive half of the parent’s stored processing energy.

If organisms divide and thereby cause the population size to exceed the predetermined maximum population capacity, organisms are selected at random to be removed from the population until the population size is equal to the maximum population capacity. Unless otherwise specified, the simulation will terminate if it reaches 100,000 time steps, the actual run time reaches 100 h, or the population has decreased to zero organisms.

### Simulation Details

In all of the simulations described above the following parameters were set as follows: all mutation rates = 0.005, food puzzle availability = infinite (except in the cellularity-dependent food puzzle transfer simulation), and the coefficient of random gene transfer = 0.5, which is multiplied by one minus the organism’s cellular impermeability value to obtain the actual probability of random gene transfer. Two different types of simulation were performed with these default settings to acquire the results presented above.

In one set of simulations (e.g., Figs. 2 and 4), otherwise identical simulations were run under four different scenarios: (A) organisms began each simulation with 0 cellularity genes and there was limitless processing energy in the environment, (B) organisms began each simulation with 0 cellularity genes and the environmental processing energy was limited to a number of energy parcels equivalent to 25% the maximum population capacity (and seeded with energy parcels containing 500 processing energy units), (C) organisms began each simulation with 3 cellularity genes and there was limitless processing energy in the environment, (D) organisms began each simulation with 3 cellularity genes and the environmental energy was limited to a number of processing energy parcels equivalent to 25% the maximum population capacity (and seeded with energy parcels containing 500 processing energy units). Each scenario was simulated at maximum population capacities of 500, 1000, and 10,000, and run three different times per population capacity value.

To acquire the data for Figs. 6 and 7, the same simulations were run, but with two alternate cellularity functions. These are *cellular impermeability* = 0.25*n* and *cellular impermeability* = 0.5^*n*^, where in both cases *n* = the number of cellularity genes. To acquire the data for Figs. 9, 10, and 12, the same simulations were run, but with the random gene transfer or energy transfer probabilities set to a constant value of either 0% or 100%, rather than being a consequence of cellularity. All of these simulations were run for 100,000 time steps, but in most figures, only the first 20,000 time steps are shown because values of population-averaged cellular impermeability and metabolic proficiency had stabilized at that point in the simulation. Each of these simulations was performed with a maximum population capacity of 1000.

To acquire the data for Fig. 8, the same simulations were run, but the acquisition of food puzzles was dependent on cellular impermeability in the same way that gene and energy transfers were in previous simulations. Specifically, In the cellularity-dependent food puzzle transfer simulation, the environmental food pool was replenished with a constant but limited supply. Organisms maintained an internal food stockpile from which to draw. This stockpile was replenished only through random transfers of food puzzles with the environmental pool according to the organism’s cellularity. If an organism’s food stockpile contained at least one food puzzle, the organism used it. If its food stockpile was empty, the organism tried again in the next step. Once an organism had a food puzzle to work on, either from the environment in our main study or from its food stockpile in the cellularity-dependent food puzzle transfer simulation, it executed the next instruction in its genome, as indicated by the position of its read head.

In a second type of simulation (Fig. 3), organisms began each simulation with 3 cellularity genes and the environment was seeded with a large but exhaustible amount of processing energy equivalent to 10,000 energy parcels containing 500 energy units each. As with the previous set of simulations, all of these simulations were run for 100,000 time steps, but only the first 20,000 time steps were shown because values of population-averaged cellularity and metabolic proficiency had stabilized by that point in the simulation. These simulations were repeated 100 times and only at the maximum population capacity of 1000.

### Quantification and Statistical Analysis

Correlation between population-averaged metabolic proficiency and population-averaged cellular impermeability was demonstrated using Pearson’s *r*. The *r* value and its associated *p* value were calculated using R.

### Directional Drift Simulations

An entirely different and significantly more simple simulation was performed to demonstrate the directional drift effect observed in Fig. 9a and c (black line), the results of which are shown in Fig. 11. In these simulations, individuals have a single value associated with them called a fidelity value. At each step in the simulation, the fidelity value determines whether the individual will mutate. The statistical mutation rate properties of this simulation are similar to those of the original model simulations. Specifically, the probability of mutating at each step in the simulation is 0.5^*x*^, where *x* is the current fidelity value of the individual. This is meant to mimic the probability of a random gene transfer to or from an organism’s genome. If an individual does mutate at a given step, then their current fidelity value is increased or decreased by 1 with equal probability. A second mutation opportunity occurs at each step with a constant probability of 0.1, which is meant to mimic the insertion or deletion mutations in the original model. Two control simulations were run at the same time as the test simulation, one in which the probability of the first mutation step was 100% (mimicking a constant value of 0% cellular impermeability) and a second in which the probability of the first mutation step was 0% (mimicking a constant value of 100% cellular impermeability). Fidelity values for each individual cannot be less than 0. To obtain the results shown in Fig. 11, one million simulations were run for the test case, and both controls, and the fidelity value was recorded at either step 1000 or 10,000.

## Electronic supplementary material

Below is the link to the electronic supplementary material.

Supplementary file1 (DOCX 12 kb)

Supplementary file2 (GIF 10466 kb)

Supplementary file3 (GIF 10531 kb)

Supplementary file4 (GIF 10463 kb)

Supplementary file5 (GIF 10315 kb)

Supplementary file6 (GIF 11390 kb)

Supplementary file7 (GIF 11117 kb)

Supplementary file8 (GIF 10530 kb)

Supplementary file9 (GIF 10319 kb)

Supplementary file10 (GIF 10609 kb)

Supplementary file11 (GIF 10284 kb)

Supplementary file12 (DOCX 1091 kb)
