# Supplementary material for: The Coevolution of Cellularity and Metabolism Following the Origin of Life
Source: J Mol Evol. 2020 Aug 18;88(7):598–617. doi: 10.1007/s00239-020-09961-1 (PMC7445158; doi:10.1007/s00239-020-09961-1)
Supplement: Supplementary file 1 — Supplementary file1 (DOCX 12 kb) [file 239_2020_9961_MOESM1_ESM.docx]

Figure S1: A movie in gif format of the change in cellularity and population size over time for the experimental scenario in which organisms began each simulation with 0 cellularity genes and there is limitless energy in the environment.

Figure S2: A movie in gif format of the change in cellularity and population size over time for the experimental scenario in which organisms began each simulation with 0 cellularity genes and the environmental energy is limited to a number of energy parcels equivalent to 25% the maximum population capacity.

Figure S3: A movie in gif format of the change in cellularity and population size over time for the experimental scenario in which organisms began each simulation with 3 cellularity genes and there is limitless energy in the environment.

Figure S4: A movie in gif format of the change in cellularity and population size over time for the experimental scenario in which organisms began each simulation with 3 cellularity genes and the environmental energy is limited to a number of energy parcels equivalent to 25% the maximum population capacity.

Figure S5: A movie in gif format of the change in cellularity and population size over time for the experimental scenario in which organisms began each simulation with 3 cellularity genes and the environment was seeded with a large but exhaustible amount.

Figure S6: A movie in gif format of the change in metabolic proficiency and population size over time for the experimental scenario in which organisms began each simulation with 3 cellularity genes and the environment was seeded with a large but exhaustible amount.

Figure S7: A movie in gif format of the change in metabolic proficiency and population size over time for the experimental scenario in which organisms began each simulation with 0 cellularity genes and there is limitless energy in the environment.

Figure S8: A movie in gif format of the change in metabolic proficiency and population size over time for the experimental scenario in which organisms began each simulation with 0 cellularity genes and the environmental energy is limited to a number of energy parcels equivalent to 25% the maximum population capacity.

Figure S9: A movie in gif format of the change in metabolic proficiency and population size over time for the experimental scenario in which organisms began each simulation with 3 cellularity genes and there is limitless energy in the environment.

Figure S10: A movie in gif format of the change in metabolic proficiency and population size over time for the experimental scenario in which organisms began each simulation with 3 cellularity genes and the environmental energy is limited to a number of energy parcels equivalent to 25% the maximum population capacity.

Figure S11. Change in population-averaged cellular impermeability and metabolic proficiency levels in different conditions of environmentally-available processing energy after the addition of a replication cost dependent on genome length. The same simulations were performed in a manner similar to those shown in Figure 1 and 3 except that an additional processing energy cost to organismal replication was applied that was equivalent to two times the genome length. Offspring organisms were not allotted any of the additional processing energy cost after replication. Results for both population averaged cellular impermeability (A) and population averaged metabolic proficiency (B) are shown for three repetitions of each of four different simulations: organisms began each simulation with 0 cellularity genes in an environment with unlimited food puzzles and processing energy (black); organisms began each simulation with 3 cellularity genes in an environment with unlimited food puzzles and processing (red); organisms began each simulation with 0 cellularity genes in an environment with unlimited food puzzles, but a limited number of processing energy parcels in the environment equivalent to 25% the maximum population capacity (green); or organisms began each simulation with 3 cellularity in an environment with unlimited food puzzles, but a limited number of processing energy parcels in the environment equivalent to 25% the maximum population capacity (blue). The results of this simulation demonstrate that imposing a reasonable, genome length-dependent energy cost on organismal replication does not alter the previously observed effects of environmentally available processing energy on the evolution of cellularity or metabolism.
