# Supplementary figures and images for: The Coevolution of Cellularity and Metabolism Following the Origin of Life
Source: J Mol Evol. 2020 Aug 18;88(7):598–617. doi: 10.1007/s00239-020-09961-1 (PMC7445158; doi:10.1007/s00239-020-09961-1)

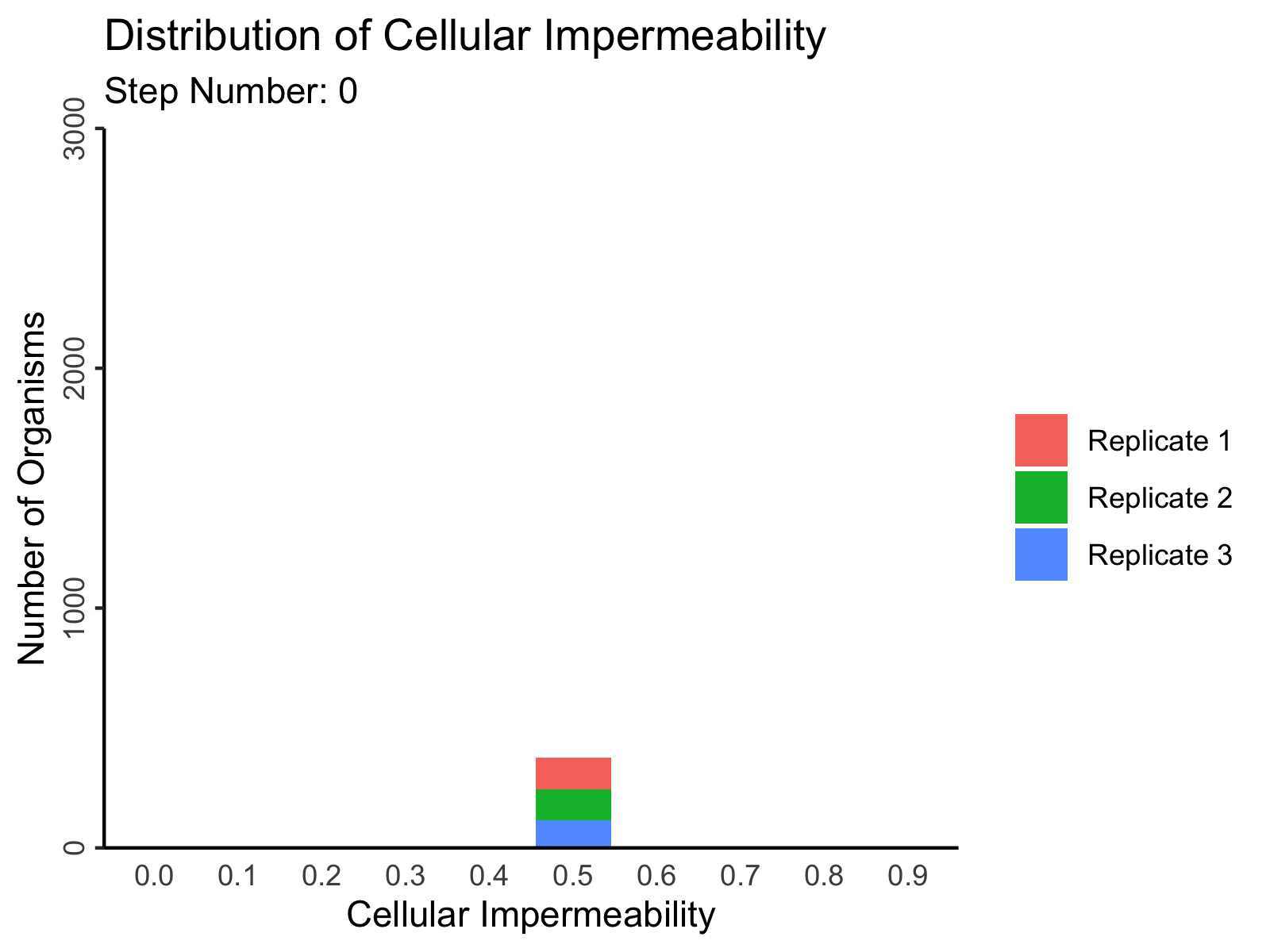

Supplement: Supplementary file 2 — Supplementary file2 (GIF 10466 kb) [file 239_2020_9961_MOESM2_ESM.gif]

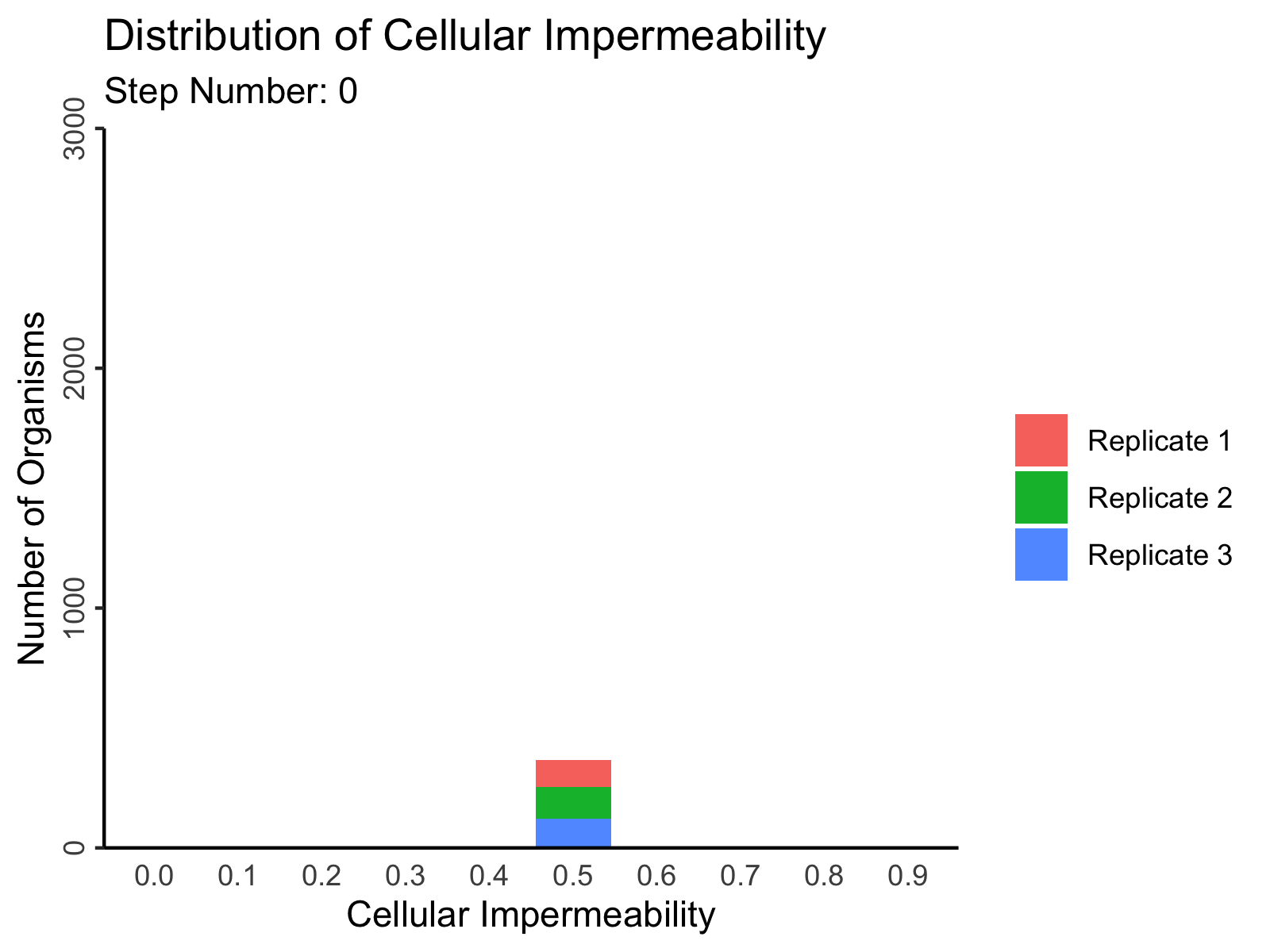

Supplement: Supplementary file 3 — Supplementary file3 (GIF 10531 kb) [file 239_2020_9961_MOESM3_ESM.gif]

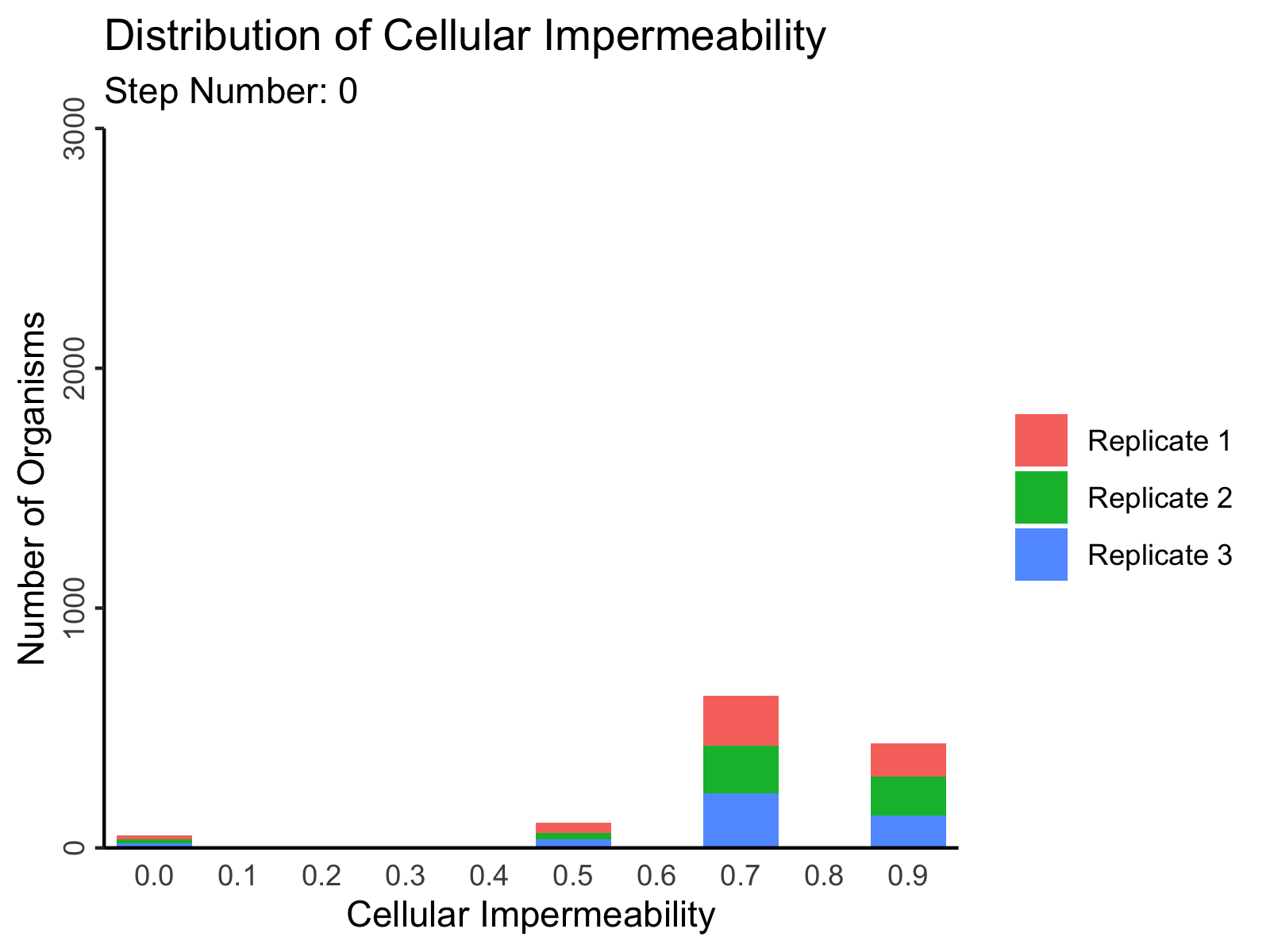

Supplement: Supplementary file 4 — Supplementary file4 (GIF 10463 kb) [file 239_2020_9961_MOESM4_ESM.gif]

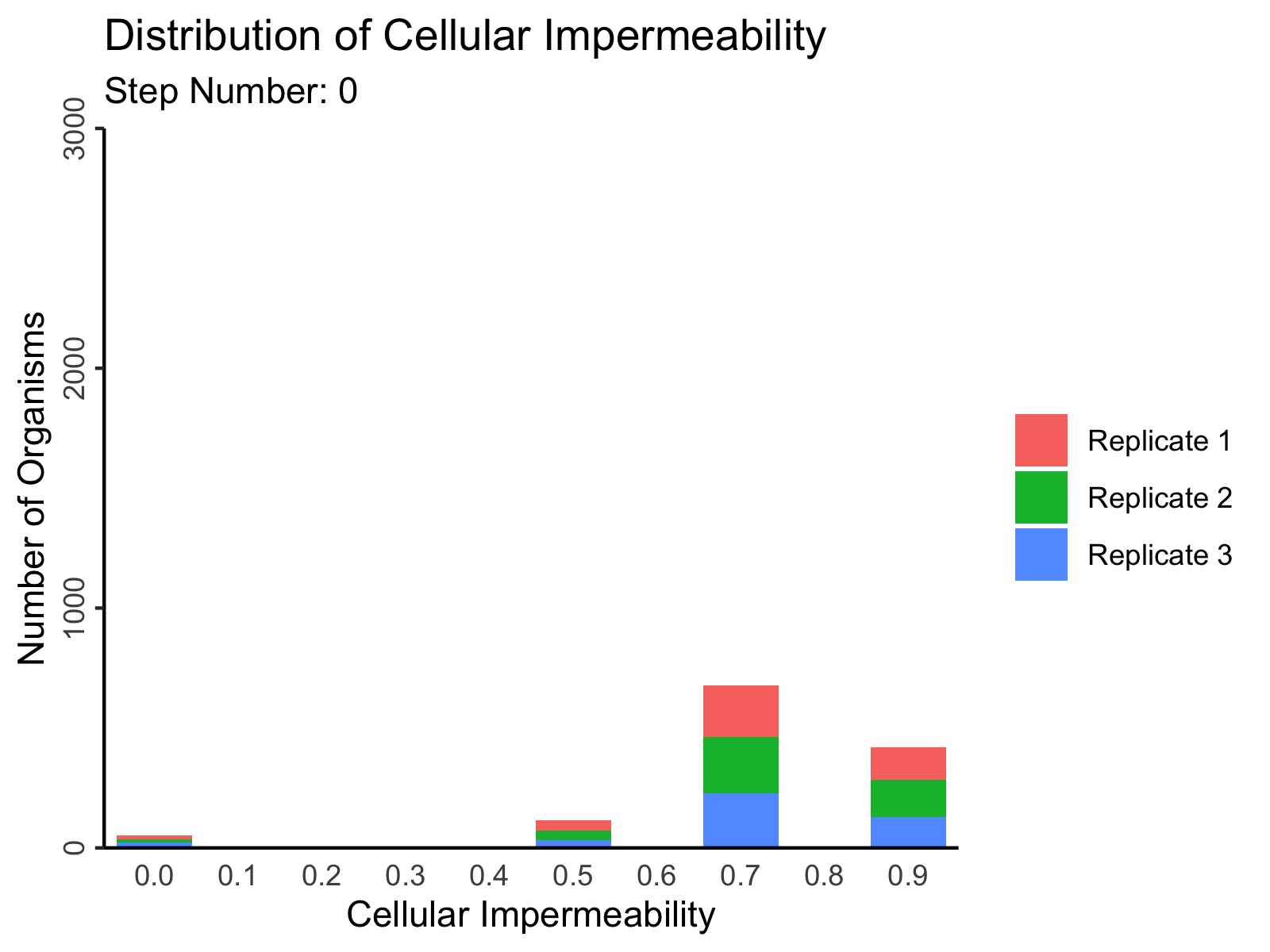

Supplement: Supplementary file 5 — Supplementary file5 (GIF 10315 kb) [file 239_2020_9961_MOESM5_ESM.gif]

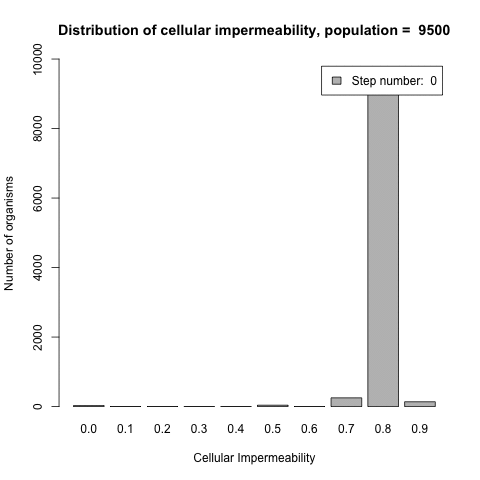

Supplement: Supplementary file 6 — Supplementary file6 (GIF 11390 kb) [file 239_2020_9961_MOESM6_ESM.gif]

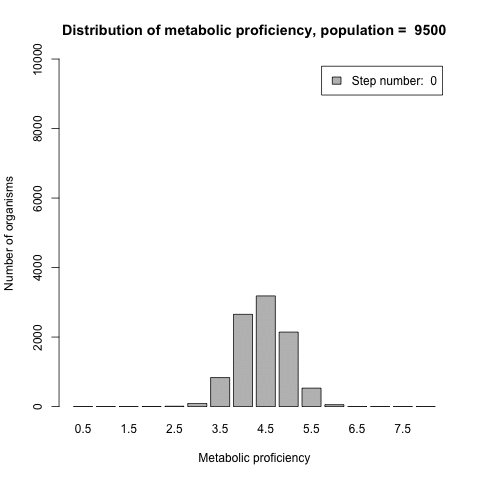

Supplement: Supplementary file 7 — Supplementary file7 (GIF 11117 kb) [file 239_2020_9961_MOESM7_ESM.gif]

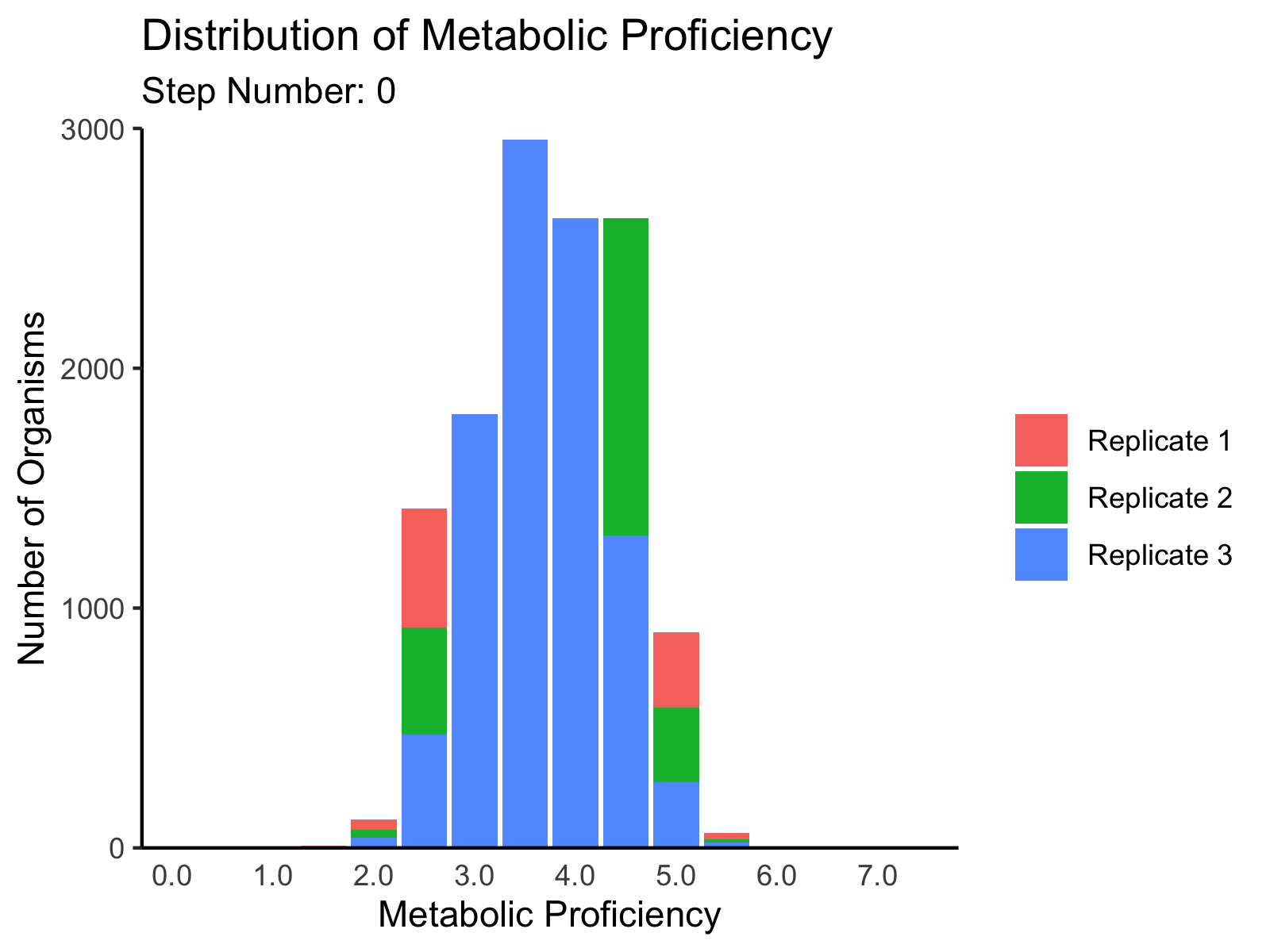

Supplement: Supplementary file 8 — Supplementary file8 (GIF 10530 kb) [file 239_2020_9961_MOESM8_ESM.gif]

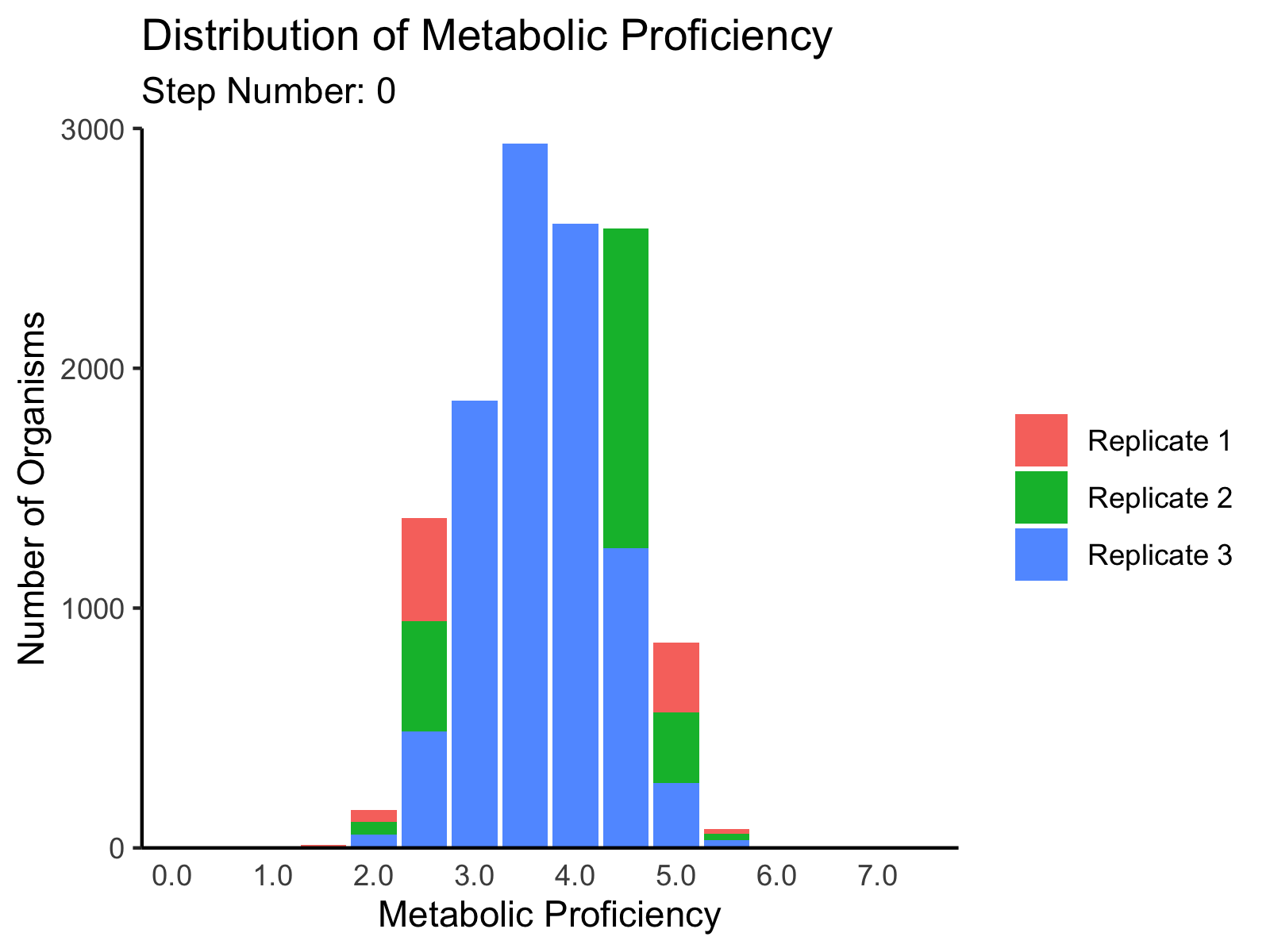

Supplement: Supplementary file 9 — Supplementary file9 (GIF 10319 kb) [file 239_2020_9961_MOESM9_ESM.gif]

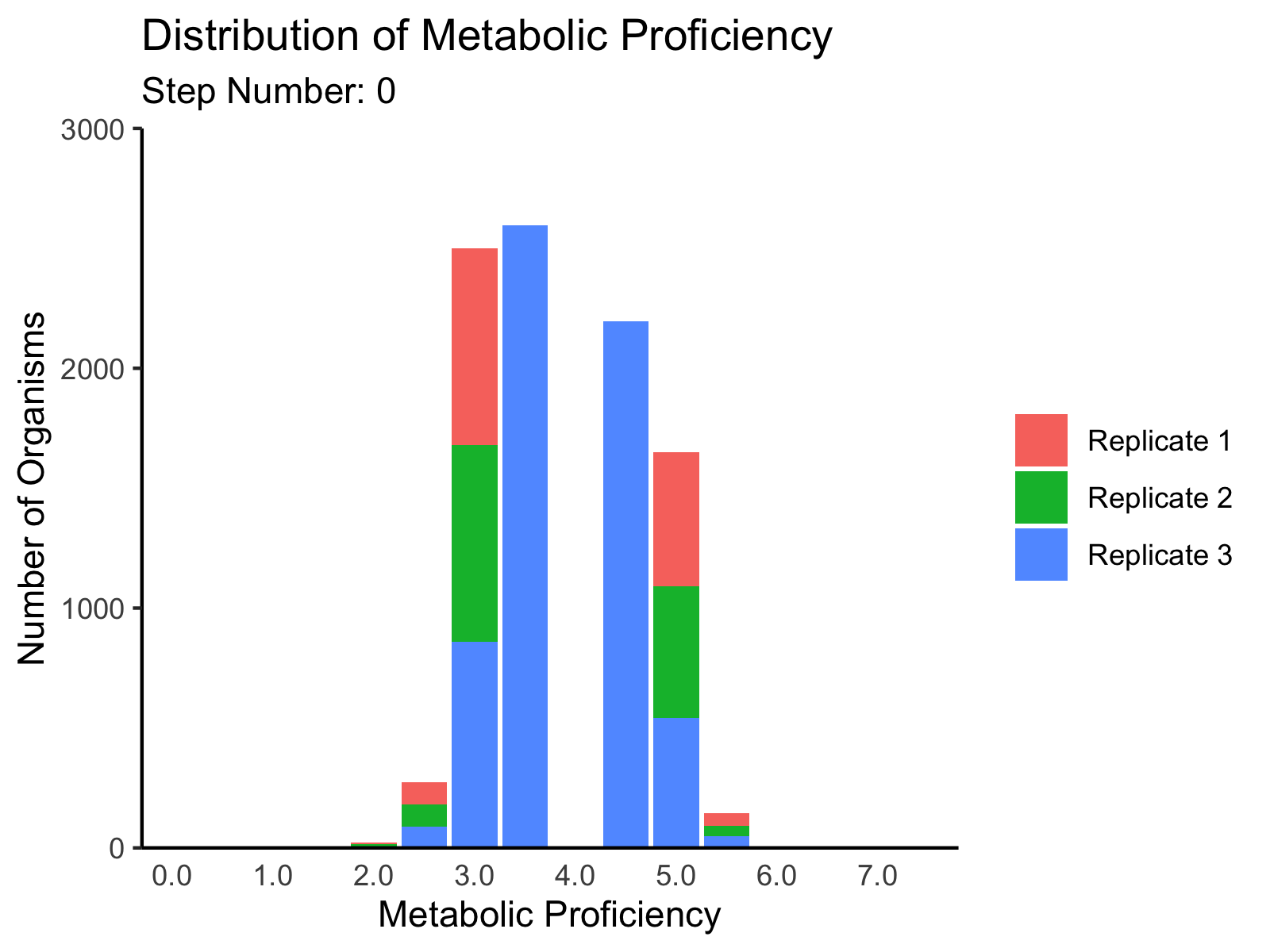

Supplement: Supplementary file 10 — Supplementary file10 (GIF 10609 kb) [file 239_2020_9961_MOESM10_ESM.gif]

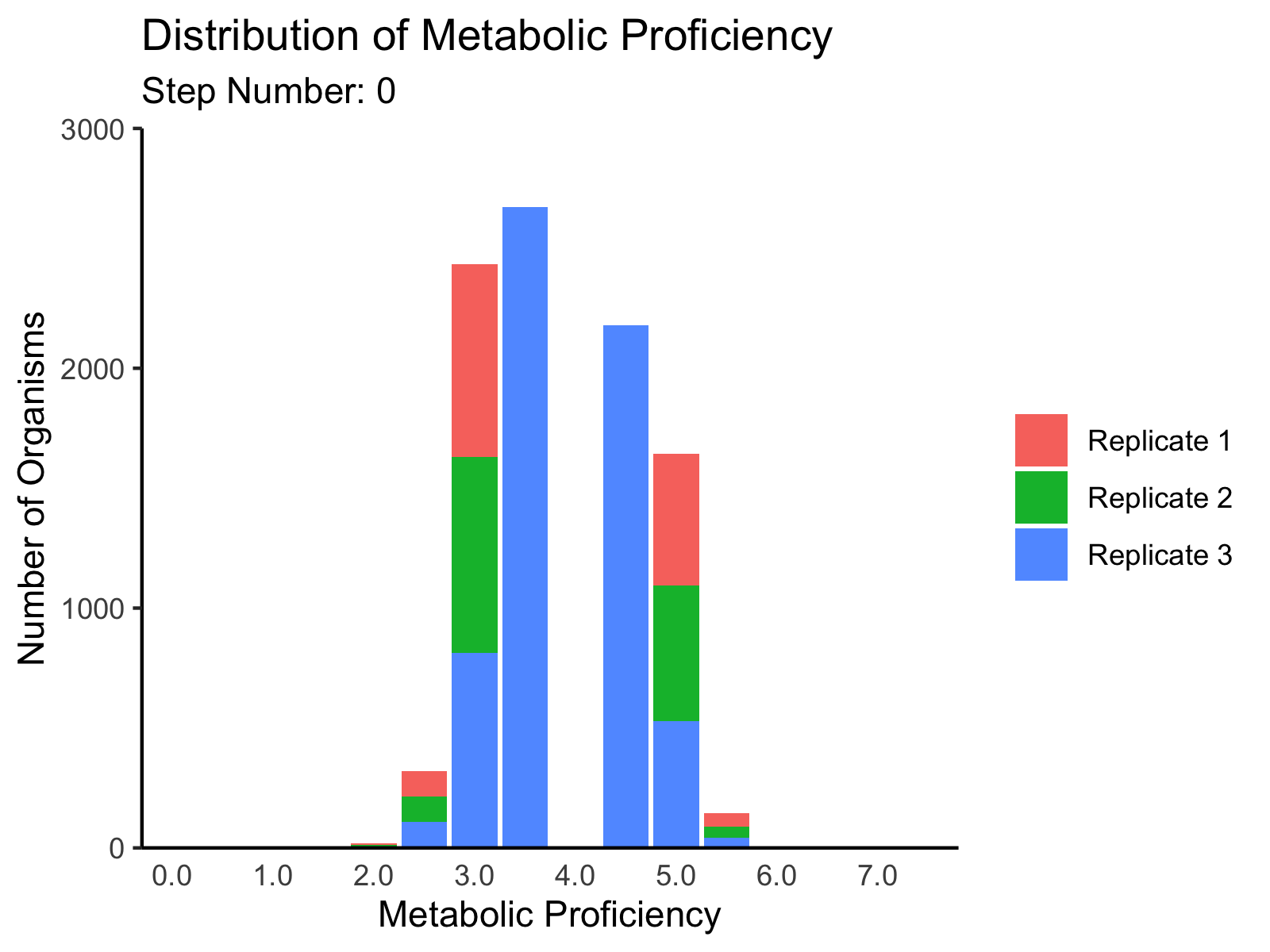

Supplement: Supplementary file 11 — Supplementary file11 (GIF 10284 kb) [file 239_2020_9961_MOESM11_ESM.gif]

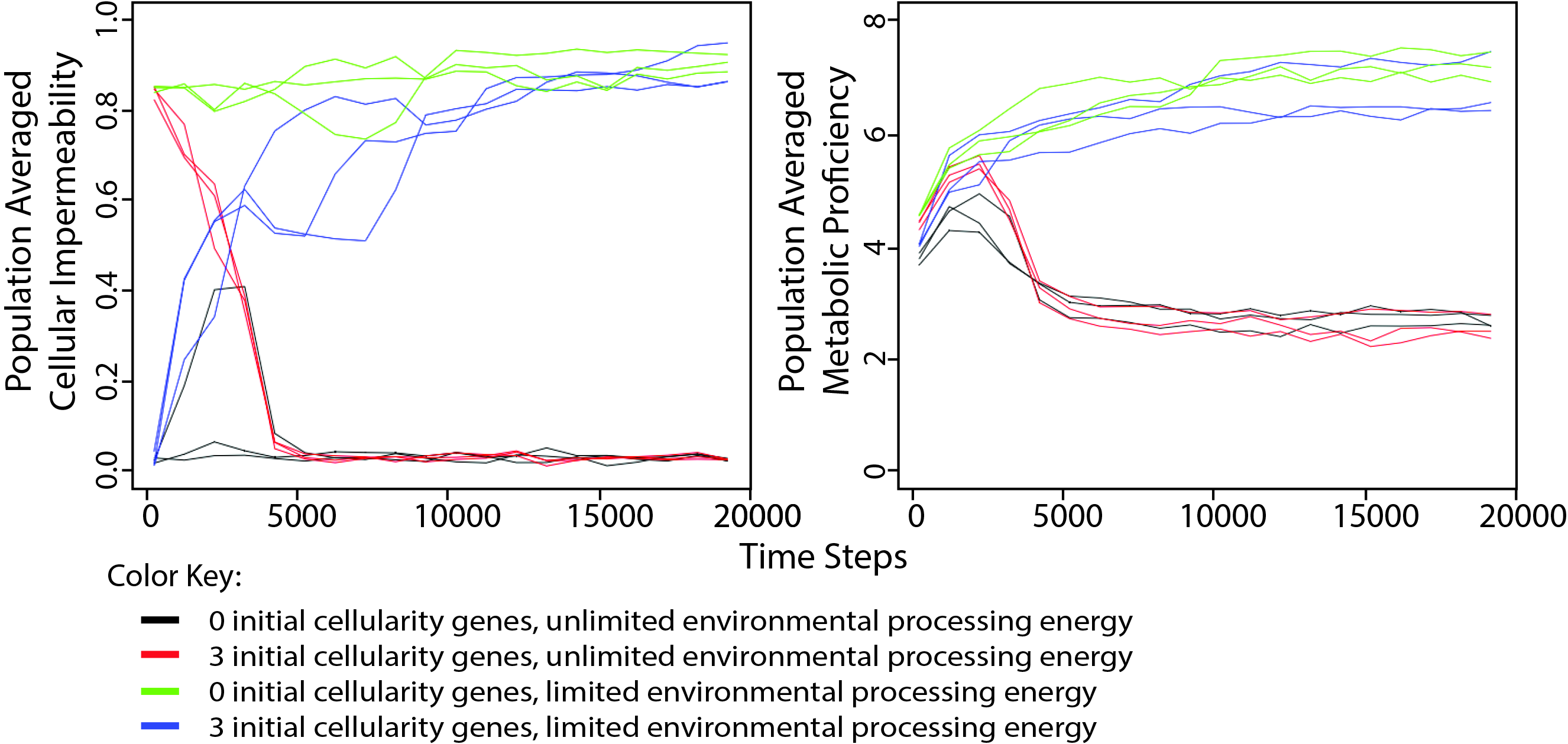

Supplement: Supplementary file 12 — Supplementary file12 (DOCX 1091 kb) [file 239_2020_9961_MOESM12_ESM.docx]
